# Phylogenetic conservatism in skulls and evolutionary lability in limbs – morphological evolution across an ancient frog radiation is shaped by diet, locomotion and burrowing

**DOI:** 10.1186/s12862-017-0993-0

**Published:** 2017-07-10

**Authors:** Marta Vidal-García, J. Scott Keogh

**Affiliations:** Research School of Biology, The Australian National University, Canberra, Australia

**Keywords:** Morphology, Modularity, Morphological integration, 3D morphology, Geometric morphometrics, Phylomorphospace

## Abstract

**Background:**

Quantifying morphological diversity across taxa can provide valuable insight into evolutionary processes, yet its complexities can make it difficult to identify appropriate units for evaluation. One of the challenges in this field is identifying the processes that drive morphological evolution, especially when accounting for shape diversification across multiple structures. Differential levels of co-varying phenotypic diversification can conceal selective pressures on traits due to morphological integration or modular shape evolution of different structures, where morphological evolution of different modules is explained either by co-variation between them or by independent evolution, respectively.

**Methods:**

Here we used a 3D geometric morphometric approach with x-ray micro CT scan data of the skull and bones of forelimbs and hindlimbs of representative species from all 21 genera of the ancient Australo-Papuan myobatrachid frogs and analysed their shape both as a set of distinct modules and as a multi-modular integrative structure. We then tested three main questions: (i) are evolutionary patterns and the amount and direction of morphological changes similar in different structures and subfamilies?, (ii) do skulls and limbs show different levels of integration?, and (iii) is morphological diversity of skulls and limbs shaped by diet, locomotion, burrowing behavior, and ecology?.

**Results:**

Our results in both skulls and limbs support a complex evolutionary pattern typical of an adaptive radiation with an early burst of phenotypic variation followed by slower rates of morphological change. Skull shape diversity was phylogenetically conserved and correlated with diet whereas limb shape was more labile and associated with diet, locomotion, and burrowing behaviour. Morphological changes between different limb bones were highly correlated, depicting high morphological integration. In contrast, overall limb and skull shape displayed semi-independence in morphological evolution, indicating modularity.

**Conclusions:**

Our results illustrate how morphological diversification in animal clades can follow complex processes, entailing selective pressures from the environment as well as multiple trait covariance with varying degrees of independence across different structures. We suggest that accurately quantifying shape diversity across multiple structures is crucial in order to understand complex evolutionary processes.

**Electronic supplementary material:**

The online version of this article (doi:10.1186/s12862-017-0993-0) contains supplementary material, which is available to authorized users.

## Background

Understanding morphological evolution, and the underlying mechanisms that generate the enormous phenotypic diversity we see, is a central aim in evolutionary biology [1–4]. Phenotypic diversity often is correlated with ecology and behaviour, especially in traits for which form and function are tightly associated due to evolutionary and ecological pressures [5–8]. However, while some clades display extensive ecological and morphological variation that is correlated with lifestyle, others retain ancestral environmental niches and conserved body shape patterns that are better explained by phylogenetic conservatism on a shared ancestral lifestyle [9, 10]. These differing patterns of diversification are best illustrated in related groups of species where one group might display more phenotypic diversification than another due to different selective pressures [4, 11]. There are many examples of this in the species-rich radiations of characiform fishes [11], gobies and cardinal fishes [12], passerine birds [13], archosaurs [14], and many others.

While diverse evolutionary processes can generate phenotypic change, morphological evolution is typically inferred from integration or co-variation among multiple traits [15]. Body shape patterns can usually be broken down into ‘modules’, which are characterized by more internal integration within them, than externally among them [15]. Therefore, each module displays a certain amount of independence from other modules and can differ developmentally, genetically, and in the way they respond to selection [15, 16]. While many phenotypic changes across a radiation are modular in this way [17], shape diversification can follow a more complex pattern of integrative co-variation between modules and show correlated morphological variation among them [15, 18]. The degree of shape-co-variation between modules is due to the interplay between morphological integration and modularity, where morphological modules evolve in concert with others and in which morphology evolves independently among different structures, respectively [15]. High morphological diversity could be correlated with modularity, as autonomy among different structural units might promote higher independent morphological changes due to the evolutionary lability necessary for adaptive shifts [19–21]. Conversely, morphological integration could be one of the causes leading to convergence among unrelated clades [22, 23]. Integration and co-variation among modules should also shape the morphological evolution of individual organisms, as some modules might be subject to strong selective pressures from the environment, whereas others might be phylogenetically constrained. Therefore, identifying the patterns of variation in each module, while accounting for integration among them, is crucial in order to study morphological evolution and the processes that might have driven it [23].

Due to the close relationship between form and function, some morphological traits are likely to be more closely linked to the ecology of an organism than others [24]. For example, Zaaf & Van Damme [25] proposed the idea of evaluating morphological differences between and within distinct modules in limbs, in relation to functional traits like locomotion, and tested it in climbing and ground-dwelling geckos. Limb shape might provide the most insight into the ecotype a species occupies, as it is closely correlated with its performance, and thus, locomotion through the environment [26, 27]. Similarly, Cornette et al. [28] looked at both the skull and mandible in shrews in order to disentangle the relationship between diet, ecological factors, and head shape evolution. On the other hand, some modules might be correlated with life history traits or not be under selection as functional traits [29]. Moreover, inferring adaptive processes by looking at the ‘wrong’ structure might be uninformative, and in some cases even misleading. Assessing morphological evolution in a group of organisms provides more valuable information when looking at a wide range of phenotypic traits, but may also increase the difficulty of data interpretation, due to complex co-variation processes between different structures.

Anuran amphibians are an ideal model group in which to investigate morphological evolutionary patterns: they display a highly derived morphology compared to other terrestrial vertebrates [30], yet their body plan has been relatively conserved since the early Jurassic [31, 32]. Despite phylogenetic constraints on their appendicular skeleton as an adaptation to saltatory locomotion [33], substantially different body shape patterns have evolved independently across several clades [34]. Frogs and toads have adapted to a wide array of extreme environments through a combination of behavioural, physiological, and morphological mechanisms. Extreme morphological shifts are usually associated with unique locomotor types, such as gliding in “flying” frogs [35], or with specialised locomotion, such as the improved swimming ability in frogs like pipids [36]. Similarly, strong shape changes are observed in burrowing frogs and toads that have adapted to desiccating conditions in arid and semi-arid environments [26, 37]. Morphological convergence in burrowing frogs has been documented across numerous clades, in both forward (head and forelimbs first), and backward (hindlimbs first) burrowing species, with backward burrowing being the most common digging type in frogs and toads (~95%), yet unique among vertebrates [38]. These diverse morphological adaptations make frogs an ideal system in which to study modularity and integration, as they relate to ecology.

The family Myobatrachidae is an old Gondwanan lineage endemic to Australia and New Guinea with its closest relatives in South America [39]. The family currently comprises 133 described species across 21 genera, accounting for 57% of the Australian frog diversity [40]. Australia’s large landmass is characterised by a wide range of biomes and has a complex history of isolation, aridification and broad climatic changes that have had a strong impact on the evolutionary processes in its biota [41]. Myobatrachid frogs are extremely diverse in ecology (from tropical rainforest dwellers to exclusive alpine species or desert-specialists; [42]), locomotion (including excellent swimmers, jumpers, hoppers, and walkers), reproductive systems (egg deposition, calls, parental care modes, etc.; [43, 44]), and also body shape patterns [45]. Thus, they stand out as a model system to examine morphological diversification patterns on a diverse and species rich radiation across a whole continent.

We sought to address three broad questions: (i) is morphological evolution similar in different body parts, (ii) do skulls and limbs show different levels of integration?, and (iii) is morphological diversity of skulls and limbs shaped by diet, locomotion, burrowing behavior, and ecology? To do this we used 3D imaging across all genera of myobatrachids, combined with geometric morphometric analyses, to discriminate the morphological integration hypothesis and the modularity hypothesis in different structures. We used 3D data from the skull and several limb bones of the appendicular skeleton (radioulna, humerus, tibiofibula and femur), and studied their shape both as a set of distinct modules and jointly as a multi-modular integrative structure. First, we sought to quantify skull and limb shape differences across representatives of all 21 genera of myobatrachid frogs by using 3D microCT scans and geometric morphometric (GM) techniques. We then addressed three major aims. First, we tested the hypothesis that evolutionary patterns and morphological disparity are similar in the two major clades of myobatrachids across different structures. We predicted that both skull and limbs followed an evolutionary pattern typical of an adaptive radiation, and that dispersion across morphospace would be correlated with species richness, with this trend being consistent across most modules. We then determined whether there were differences in dispersion and direction of shape diversification in skulls and limbs, and whether morphological evolution acts independently in each module, or if there was some integration across different structures. We predicted a high degree of morphological integration, especially among limb modules, due to selective pressures derived from environmental correlates and associated adaptations such as burrowing behavior and locomotion. Finally, we tested for relationships between morphology and burrowing, locomotion, and environment. We predicted that form would be correlated with function, i.e. ecology, locomotion, and burrowing behavior would have been key drivers in shaping morphological evolution on the limbs, whereas head shape would be more phylogenetically conserved due to a lower functional pressure imposed by the environment.

## Methods

### Study samples and morphological data

This study is based on 41 ethanol-preserved specimens from 21 species of the Australo-Papuan myobatrachid frog radiation. Sampling covered all genera from this family, and with the exception of the monotypic *Spicospina flammocaerulea* where only one specimen was available, we used two representative specimens of the same species per genus as a previous study across all myobatrachid species showed high morphological conservatism within genera [45]. Species and voucher number details are presented in Additional file 1: Appendix S1. Since sexual dimorphism is known in some myobatrachid species (e.g. *Adelotus brevis*), we only sampled adult females in order to avoid morphological differences due to sexual dimorphism. We gathered data for burrowing behavior from several sources [42, 46] and classified each species into three categories based on the type of burrowing: (a) forward burrowers which use their forelimbs, (b) backwards burrowers which use their hindlimbs, and (c) non-burrowers. Locomotion information was gathered from Anstis (2013) and Cogger (2014), and locomotor mode categories were defined according to basic characteristics of their stride: (a) walkers are species that are strictly walkers or crawlers, (b) hoppers are species that can only hop, or hop and walk, and not jump (an average jumping distance that is less than five times their body length), and (c) jumpers/swimmers are species that can jump and/or swim (whose average jumping distance is greater than five times their body length and are proficient swimmers). Even though some genera display multiple states for burrowing and locomotor modes, the analyses were performed using the state present on the selected species. Data for habitat type or ecoregions was gathered taking into account each species’ distribution and the seven main ecoregions in Australia [42, 47]: (a) tropical and subtropical moist broadleaf forests, (b) temperate broadleaf and mixed forests, (c) tropical and subtropical grassland, savannas and shrublands, (d) temperate grasslands, savannas and shrublands, (e) montane grasslands and shrublands, (f) mediterranean forests, woodlands and shrubs, and (g) deserts and xeric shrublands. Dietary information [48–58] was gathered for all species in this study (except for the little-known species *Spicospina flammocaerulea,* for which we inferred diet from its close relatives and based on similarities in other life-history traits), which was classified into two categories: (a) generalists have multiple taxa represented in their diet, regardless of their size) and (b) specialists only feed on certain taxa (mostly termites and ants). Data on burrowing behavior, locomotion, diet, and ecoregions is summarized on Additional file 1: Table S1. All morphological data was gathered using three different X-ray micro-CT scanners, depending on the size of the individual frog: Skyscan 1174 (Bruker micro-CT) for small frogs, MicroXCT-400 (Xradia system) for intermediate sized frogs, and a custom-made double-helical x-ray micro CT scanner from the Australian National University for the larger specimens. The settings for each CT scanner were as follows: Skyscan 1174–40 kV source voltage, 800 μA source current, voxel size of 32.47 μm, 0.7° rotational step, 1.6 s exposure time, and 360° rotational angle scanning. The acquired images (angular projections) were reconstructed into a virtual stack of 2D cross-section slices using the NRecon (Skyscan) software interface. Xradia MicroXCT-400 - 50 kV, 360° rotational angle scanning, 2 s exposure, and voxel size of 49.13 μm. Acquired images were reconstructed in the MicroXCT and exported to a virtual stack of 2D cross-section slices (8-bit BMP format) using Avizo software system (version 8.0, Mercury Computer Systems, Inc., Germany). Custom-made double-helical x-ray micro CT - 80 kV, 100 μA, voxel size of 43 μm, using a 0.3 mm Al filter, 3.4 s exposure, and 0.143° rotational step, resulting in 2520 angular projections. This RAW data was also then reconstructed into 2D cross-section slices (NC format). Each stack of reconstructed images was then converted into 3D data, using the volume-rendering software *Drishti* [59].

### Shape analyses

Skull and limbs’ bone shape differences were identified using geometric morphometric (GM) methods. We used rendering software *Drishti* [59] in order to digitise 3D landmarks of the skull and limb bones, and also sliding semilandmarks on limb bones (Additional files 2 and 3). We then averaged each dataset of morphometric data by species with *geomorph* [60], in order to allow analyses in a phylogenetic context and focus on morphological variation among genera and clades. We also performed GM analyses with all raw data sets before taking species means to ensure that interspecific variation was greater than intraspecific variation. Each data set was subjected to a generalised Procrustes sumperimposition fit with the package *geomorph* [60–62]. We performed a Principal Component Analysis (PCA) on the projected Procrustes coordinates into the tangent space for each set of morphological data. Each data set of GM data was analysed separately, but also joined, considering each long bone as a distinct module. To do so, we translated and rigidly rotated all landmarks and semi-landmarks from each data set using a newly developed Rigid Rotation equation, with the R package *ShapeRotator* [63]. This allowed us to set up all the different modules in the same position, angle and torsion and thus allow us to analyse different mobile structures as a whole (as modules would be in the same position relative to each other). We then analysed shape and size differences across all genera in each module and also in each different group of modules: (a) forelimbs (H + RU), (b) hindlimbs (F + TF) and limbs (H + RU + F + TF). In order to test our modularity and morphological integration hypotheses we also analysed morphological co-variation between: (a) skull and the four modules in the limbs (H + RU + F + TF), (b) co-variation between forelimbs (H + RU) and hindlimbs (F + TF), (c) whithin each limb, so between radioulna and humerus in forelimbs and in femur and tibiofibula in hindlimbs, and among different modules within the skull (Additional file 1: Table S2).

### Statistical analyses

In order to investigate patterns of morphological evolution across the myobatrachid frog family we used a phylogeny for the group based on two mtDNA genes (ND2 and 12S) and two nDNA loci (Rag1 and Rhodopsin). This is the same phylogeny we used in a previous study of shape evolution in these frogs ([45]; toplogy available on dryad: https://datadryad.org/resource/doi:10.5061/dryad.1vb63) We used the R package *ape* [64] to prune this tree to only include the species used in this study, and to produce an ultrametric tree with branch lengths approximating proportions of their total age. The resulting phylogeny was projected onto morphospace (previously obtained through PCA of the Procrustes coordinates) with *geomorph* [60] to visualise shape differences in a phylogenetic context for each of the data sets [11, 65, 66]. We also used thin-plate spline (TPS) deformation grids to visualise shape changes in the skull in the three dimension (TPS grids for x, y and x, z) using *geomorph* [60]. To test for the strength of phylogenetic signal in our shape data we calculated the *K*-statistic’s generalization for multivariate data (*K*_*mul*t_; [67]) with *geomorph* [60] on the Procrustes-aligned coordinates for each GM data set. We considered a strong phylogenetic signal (*K*_*mult*_ presenting values grater 1) as the null hypothesis which means that closely-related taxa would occupy similar regions in morphospace [68]. We tested which evolutionary model of phenotypic evolution best fits our data, for both the skull and the limbs (all four limbs bones) shape data sets, using the R packages *geiger* [69] and *ouch* [70] in the first five Principal Components. Since the results were not congruent among each PC, we decided to take a multi-variate approach using the R package *mvMORPH* [71], which allows complex model fitting in multivariate data. We tested the best fit for multiple models of morphological evolution in the first ten PCs of both the skull and the limbs data sets, and selected diet as a shift since it was found to be correlated to shape differences in both skulls and limbs. The models tested were: BM (Brownian Motion), BM two rates (based on diet), OU (Ornstein–Uhlenbeck), OU with two adaptive optima, EB (Early Burst), and twelve different models with a shift from two different processes at a given point in time in which some had independent rates on each time slice (Table 1).

**Table 1:** Summary statistics for the fit of models of phenotypic evolution in the multivariate shape datasets of Skull and limbs (all four limb bones’ analysed together): maximum likelihood estimate (ln L), sample-size corrected Akaike’s Information Criterion (AICc), and Delta AICc (∆AICc, difference between a model and the model with the lowest AICc)

Variable	SKULL			LIMBS		
	ln L	AlCc	∆AlCc	ln L	AlCc	∆AlCc
BM1	395.321	−851.309	100.238	586.680	−1234.026	117.504
BMM	437.403	−925.206	26.341	645.680	−1341.761	9.769
OU1	414.072	−878.544	73.003	611.689	−1273.778	77.752
OUM	427.652	−904.941	46.606	629.454	−1308.544	42.986
EB	392.314	−844.888	106.659	583.708	−1227.677	123.853
BM_EB	401.074	−862.409	89.138	586.723	−1233.707	117.823
EB_BM	400.259	−860.780	90.767	586.626	−1233.512	118.018
BM_EBi	447.326	−944.969	6.578	649.317	−1348.950	2.580
EB_BMi	**450.615**	**−951.547**	**0**	**650.606**	**−1351.530**	**0**
BM_OU	445.226	−940.852	10.695	620.337	−1291.075	60.455
OU_BM	425.734	−901.867	49.680	618.049	−1286.499	65.031
BM_OUi	448.231	−943.881	7.666	645.977	−1339.372	12.158
OU_BMi	330.765	−708.949	242.598	404.802	−857.024	494.506
EB_OU	408.027	−866.371	85.177	598.161	−1246.638	104.892
OU_EB	430.634	−911.585	39.962	617.650	−1285.623	65.907
EB_OUi	446.735	−940.855	10.692	649.460	−1346.304	5.226
OU_EBi	332.339	−712.063	239.484	407.160	−861.704	489.826

We tested the fit of the following evolutionary models: BM1 = Brownian Motion (unique rate), BMM = Brownian Motion (multiple rates), EB = Early Burst, and 12 evolutionary models with shifts from one model to another (e.g. BM_EB = shift of a BM to EB process, EB_BM = shift of EB to BM, BM_EBi = BM_EB with independent rates, EB_BMi = EB_BM with independent rates, etc.). Analyses were performed in R using the functions *mvBM(), mvOU(), mvEB()* and *mvSHIFT*() from the R package *mvMORPH* (Clavel et al., [71])

We tested for evolutionary allometry by performing a regression of shape variation on size variation among different species in a phylogenetic context [72]. In order to test whether shape variation was correlated to burrowing behavior, locomotor mode, or ecoregion, we performed phylogenetic ANOVAs using the function procD.pgls() in *geomorph* [60] on Procrustes-aligned coordinates from each GM data set for diet, locomotor mode, burrowing behaviour, and ecology (bioregions). We also performed a phylogenetic ANOVA with all the factors, and factorial phylogentic ANOVAs with pairs of factors and their interactions. We performed a Mantel test using the R package *vegan* [73] to test whether there was an association between the species distribution in the skull and the limbs shape data sets, using a Spearman’s Rank correlation coefficient. Finally, we also tested for morphological disparity among the main four clades and subfamilies in the myobatrachid frog radiation in each GM data set, in relation to the number of genera per clade and the age of each clade. We used Procrustes variance (mean squared Procrustes distance of each genera from the mean shape of their clade) as a measure of morphological disparity which was calculated using *geomorph* [60]. Finally, we used two-block partial least squares (PLS) analysis in order to quantify shape co-variation between different structures, using *geomorph* [60]. We performed two-block PLS analyses between: a) skull and the overall limb shape (RU + H + TF + F), b) forelimbs (RU + H) and hindlimbs (TF + F), c) radioulna and humerus, and d) tibiofibula and femur. All two-block PLS analyses were performed on the Procrustes-aligned coordinates from each GM data set. We also assessed phylogenetic morphological integration between all these modules using the function phylo.integration() in *geomorph* [60].

## Results

### Size and shape variation

Evolutionary allometry did not account for a significant amount of variance on skull shape: the multivariate regression of Procrustes-aligned coordinates (shape) on log-transformed centroid size (size) demonstrate that only 6.77% of the total shape variation is correlated to size variation (*p* = 0.23). Similarly, evolutionary allometry of limb bones was also low: only 4.29% of the total variance in total limb shape (RU + H + TF + F; *p* = 0.15) was correlated to size changes, 3.76% for forelimb shape (RU + H; *p* = 0.19), and it was slightly higher for hindlimb shape, with size correlates explaining 10.7% of the variance in shape (TF + F; *p* = 0.03). Given the small impact of size on shape variation we performed further analyses on the raw morphometric data sets without removing the allometric effects.

**Fig. 1:**
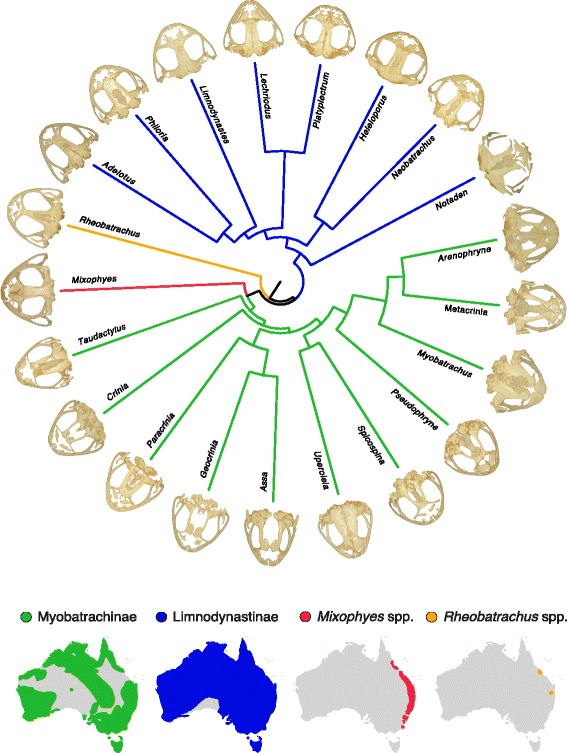
Dorsal view of skull diversity across all genera of myobatrachid frogs. The four maps display the distribution across Australian of each of the four main clades within the myobatrachids

**Fig. 2:**
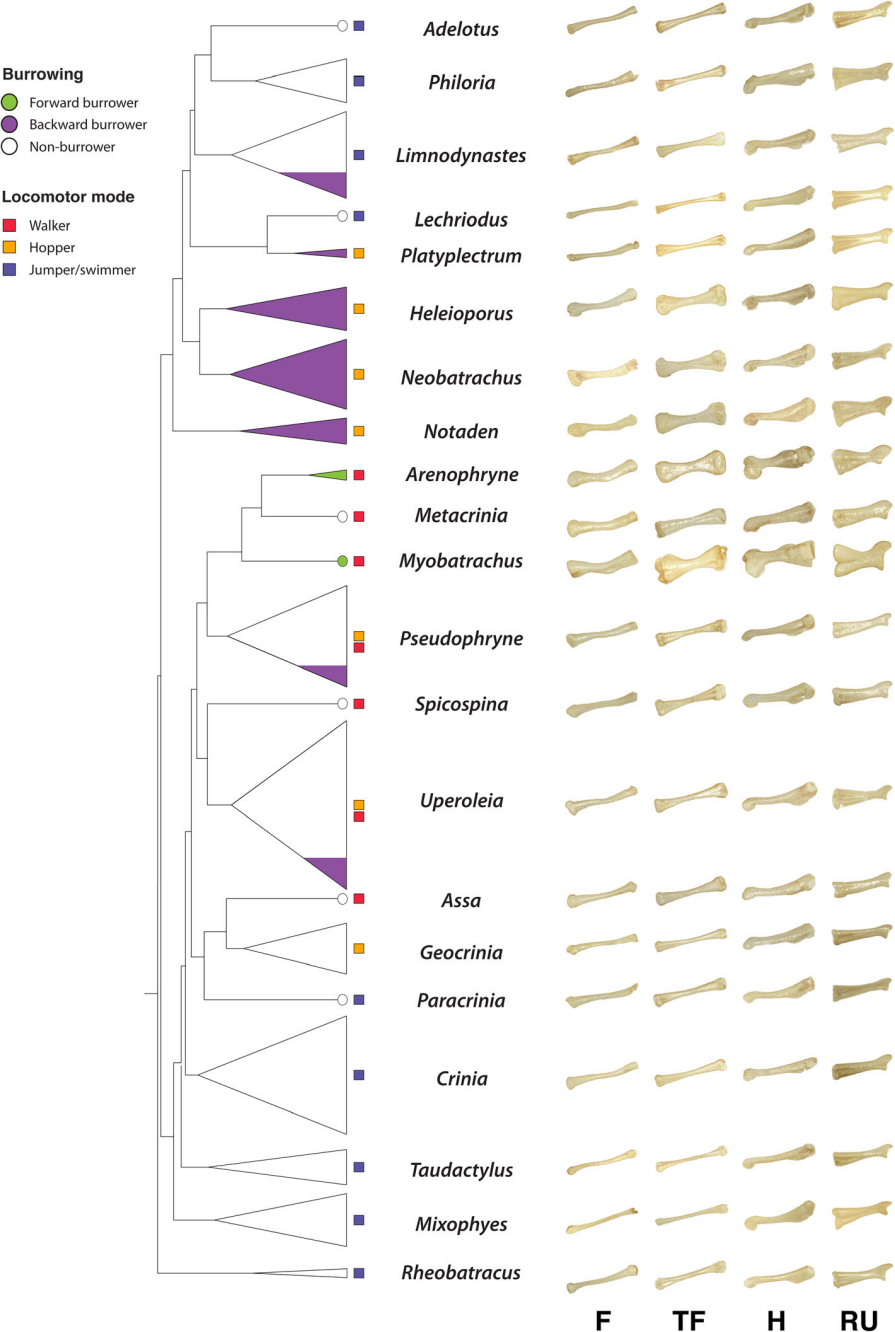
Shape diversity of limb bones in each genera of myobatrachid frogs: femur (F), tibiofibular (TF), humerus (H), and radioulna (RU). Branches on each genera have been collapsed while retaining information on the species richness of each genus. The legend depicts the three burrowing modes (forward, backward, and non-burrower) and locomotor modes (walker, hopper, and jumper/swimmer). Clades with only few species adapted to fossoriality have been indicated in the figure (*Limnodynastes* spp., *Pseudophryne* spp., and *Uperoleia* spp.)

**Fig. 3:**
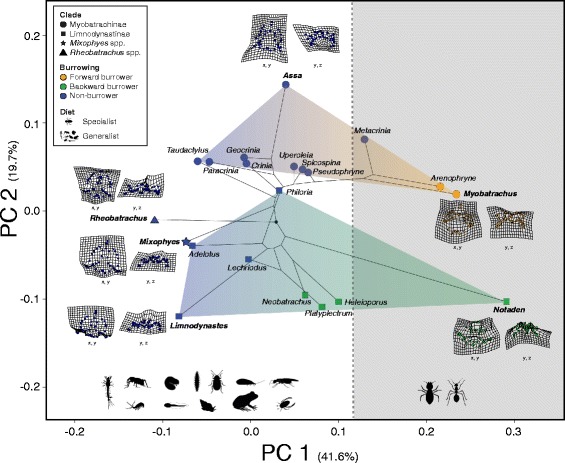
Phylomorphospace of PCA values on skull shape variation based on species means, using the R package *geomorph*. Each clade is depicted with a different shape, while burrowing behavior is indicated by different colouring. The two main different diet types (specialist and generalist) are also indicated by a schematic of each type and background colouring. Thin-plate spline (TPS) deformation grids are displayed to indicate extreme variation on skull shape among different species (names in bold), and only species from the outer limits of the morphospace are depicted

We depict skull shape variation and shape diversity across the four limb bones in Figs. 1 and 2, respectively. In the skull shape data set, the first five principal components (PC) accounted for 82.23% of the total variance (Additional file 1: Table S3), with PC_skull_ 1 and PC_skull_ 2 explaining 41.58% and 19.72% of morphological variation, respectively. The primary axis of variation (PC_skull_ 1) corresponded to width and height of the cranium, and separated burrowing species (both forward burrowers and backward burrowers) and non-burrowing species (Fig. 3). The second axis of variation (PC_skull_ 2) mainly corresponded to variation in the shape of the snout (from pointy to very rounded snouts), and clearly grouped the main two clades in different regions of the morphospace (Fig. 3). Cranium variation is also depicted in Fig. 1 through TPS grids of individuals that present the most extreme morphological variation from the consensus cranium shape.

**Fig. 4:**
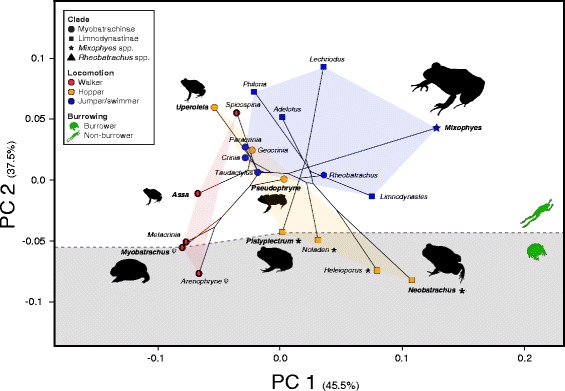
Phylomorphospace of PCA values on overall limb shape variation based on total shape variation of radioulna, humerus, and femur, and generated with geomorph. Different shapes depict each of the four clades, while colour represents locomotor mode: Walker, Hopper, and Jumper/Swimmer. Burrowing behavior is also indicated in the figure by a schematic of each type (burrower and non-burrower) and background colouring, and the signs ψ and * indicate whether the burrower is forward-burrowing (head and forelimbs first) or backward-burrowing (hindlimbs first), respectively. Outlines of overall body shape are displayed in species with the most extreme limb shape variation

For radioulna (RU) shape variation, the first five PCs explained 78.06% of the variance (Additional file 1: Table S3), with PC_RU_ 1 representing 57.99%, and being mostly correlated with arching on the diaphysis of the radioulna (ranging from extremely curved and constricted radioulnas in the medial part of the diaphysis to an almost straight radioulnas). PC_RU_ 2 only added an additional 7.23% (Additional file 4: Figure S1a), and was correlated with the shape of the epiphysis. The first five PCs of the humerus (H) data set accounted for 76.79% of the overall variance (Additional file 1: Table S3), with PC_H_ 1 representing 39.38%, and PC_H_ 2 23.14%, mostly accounting for relative size of the deltoid tuberosity and robustness of the whole humerus (Additional file 4: Figure S1c). On the joined data set of RU and H, the first five PCs explained 82.6% of the total shape variability (Additional file 1: Table S3), with PC_RU+H_ 1 accounting for 47.22% of the variance and PC_RU+H_ 2 another 12.24%, and most of the morphological variability represented robustness of both humerus and radioulna, and the length of the radioulna relative to the humerus (Additional file 4: Figure S1e). On the hindlimb bones data sets, shape variation was mostly accounted within the first five PCs, with 94.82% of the total variance in tibiofibula (TF) and 97.45% in femur (F; Additional file 1: Table S3). The first axis of variation in the TF data set (PC_TF_ 1) explained most of the morphological variation as it represented 62.01% of the overall variance (Additional file 1: Table S3) and was highly correlated with the robustness of the tibiofibula and the degree of constriction in the medial par of the diaphysis. PC_TF_ 2 only added an additional 13.58% (Additional file 4: Figure S1b). On the F data set, PC_F_ 1 explained 81.23% of the total morphological variance, while PC_F_ 2 only added an additional 9.88% (Additional file 4: Figure S1d), and most of the morphological variance was correlated with the degree of arching in the medial part of the diaphysis. On the joined data set of TF and F, the first five PCs accounted for 94.72% of the variance (Additional file 1: Table S3), with PC_TF+F_ 1 representing for 75.34% and PC_TF+F_ 2 an additional 9.77% (Additional file 4: Figure S1f). In contrast with the TF and F data sets, the morphospace hindlimb shape axes (PC_TF+F_ 1 and PC_TF+F_ 2) were mostly correlated with the robustness of both the femur and tibiofibula, the amount of arching observed in the femur, the degree of constriction in the medial part of the diaphysis, and the length of the tibiofibula relative to the femur. Finally, in the overall limb bones shape data set (radioulna + humerus, + tibiofibula + femur), the first five PCs accounted for 91.19% of the morphological variation, with PC_limbs_ 1 representing 45.46% of the variance and PC_limbs_ 2 an additional 37.47% (Fig. 4). Most of the shape changes in the first two axes were associated with general robustness of all four bones, and were correlated with locomotor mode: walker species displayed the most negative values in both PC_limbs_ 1 and PC_limbs_ 2 and occupied distinct regions in the morphospace, while hoppers and jumper/swimmer species overlapped and usually displayed neutral or positive values in both axes.

### Patterns of morphological evolution in heads and limbs

We found strong phylogenetic signal on the skull Procrustes-aligned coordinates with *K*_mult_ values equivalent or greater than 1, and this was significant for the skull, femur, tibiofibular, and limbs (RU + H + TF + F; Additional file 1: Table S4). This means that more closely related species to resemble each other under a Brownian Motion process. The fitting of evolutionary models to univariate data (first five PCs) in both skull and the limb (all four limbs bones) datasets supported different models for each PC (Additional file 1: Table S5). Tests for the best fitting model of phenotypic evolution in multivariate data (first ten PCs) showed support for the same complex process in both skulls and limbs: a model of Early Burst followed by a Brownian Motion process with two different rates based on diet (Table 1). Morphological disparity of skull shape was quite similar in the two most species-rich clades, with Procrustes variance (Proc_var_) of 0.022 in Myobatrachinae, and Proc_var_ = 0.030 in Limnodynastinae. In the limbs (RU + H + TF + F), morphological disparity was higher in Limnodynastinae (Proc_var_ = 0.006) than Myobatrachinae (Proc_var_ = 0.003). In forelimbs disparity was higher in Myobatrachinae (Proc_var_ = 0.007) than Limnodynastinae (Proc_var_ = 0.003). In each forelimb module separately, morphological disparity of the radioulna was higher in Myobatrachinae (Proc_var_ = 0.007; Proc_var_ = 0.002 in Limnodynastinae), and also in the humerus (Proc_var_ = 0.005 in Myobatrachinae; Proc_var_ = 0.002 in Limnodynastinae). Procrustes distances in both clades were equal in hindlimbs (TF + F; Proc_var_ = 0.004), higher in the femurs of Limnodynastinae (Proc_var_ = 0.004 in Myobatrachinae; Proc_var_ = 0.006 in Limnodynastinae), and higher in the tibiofibula of Myobatrachinae (Proc_var_ = 0.013 in Myobatrachinae; Proc_var_ = 0.009 in Limnodynastinae). The Mantel test performed on the dissimilarity matrices extracted from the PC components of skull and limbs shape data sets was not significant (*r* = −0.048, *p* = 0.678), supporting the null hypothesis that there is no association between the species distribution in the skull and the limb morphospace.

### Testing morphological integration and modularity hypotheses

The two-block partial least squares (PLS) analysis between skull and overall limb shape (RU + H + TF + F) indicates that there was slight morphological integration between head and all four limbs (r-PLS = 0.685, *p* = 0.011; r-PLS = 0.694, *p* = 0.013 after phylogenetic correction), suggesting semi-independent morphological evolution. However, this result does not hold when we look at the relationship between the head and the fore and hindlimbs separately: morphological co-variation was much higher when assessed independently on only head and forelimb (r-PLS = 0.923, *p* < 0.001; r-PLS = 0.909, *p* = 0.001 after phylogenetic correction), and even higher on head and hindlimb (*r* = 0.983, *p* = 0.002; r-PLS = 0.946, *p* = 0.018 after phylogenetic correction). Morphological integration between forelimbs (RU + H) and hindlimbs (TF + F) was moderate (r-PLS = 0.767, *p* < 0.001), but it was much higher after correcting for phylogenetic effects (r-PLS = 0.897, *p* = 0.001) Shape co-variation between the two modules in hindlimbs (F + TF) was extremely high (r-PLS = 0.968, *p* < 0.001), even after considering phylogenetic correlates (r-PLS = 0.976, *p* = 0.001). Similarly, the two-block PLS on the forelimbs was also high, supporting strong morphological integration between humerus and radioulna (r-PLS = 0.925, *p* < 0.001), even after phylogenetic correction (r-PLS = 0.932, *p* < 0.001). Thus, these results suggest that selective pressures acted on the two modules of hindlimbs (F + TF) and forelimbs as if it was a single integrative structure, but there was certain degree of independence between fore and hindlimbs. All the two-block PLS analyses among different modular partitions within the skull (both raw and taking phylogenetic relationships into account) displayed high levels of integration (Additional file 1: Table S5 and Additional file 5: Figure S2), pointing out that morphological features in the different substructures within the skull have evolved in concert.

### Ecology, locomotion and burrowing behaviour

Phylogenetic ANOVAs performed on Procrustes-aligned coordinates of the skull data set were statistically significant for diet (F_20,1_ = 6.058, *p* = 0.001). Similarly, they were also significant for burrowing (F_20,2_ = 2.806, *p* = 0.021), and locomotor modes (F_20,2_ = 3.208, *p* = 0.001). Conversely, they were not significant for the broad eco-regions based on Australian biomes (F_20,4_ = 1.233, *p* = 0.235). In the phylogenetic ANOVA with the three significant factors (diet + burrowing + locomotion), only diet was significant (F_20,1_ = 3.184, *p* = 0.005). In the factorial phylogenetic ANOVA between burrowing and locomotion, neither the factors (F_20,2_ = 3.111, *p* = 0.157 and F_20,2_ = 1.952, *p* = 0.158, respectively) nor the interaction (F_20,1_ = 1.053, *p* = 0.316) were significant. In the factorial phylogenetic ANOVA between burrowing and diet, both diet (F_20,1_ = 3.368, *p* = 0.003) and the interaction (F_20,1_ = 2.373, *p* = 0.006) were significant, whereas burrowing was not (F_20,2_ = 1.551, *p* = 0.074). Finally, in the factorial phylogenetic ANOVA between diet and locomotion, both factors (F_20,1_ = 7.629, *p* = 0.001 and F_20,2_ = 2.855, *p* = 0.018, respectively) and the interaction (F_20,1_ = 2.304, *p* = 0.003) were significant.

On the combined limb GM data set (RU + H + TF + F) phylogenetic ANOVAs, burrowing (F_20,2_ = 3.113, *p* = 0.028), locomotion (F_20,2_ = 2.848, *p* = 0.012), and diet (F_20,2_ = 3.219, *p* = 0.006) had a significant effect on overall limb shape, whereas ecorregions (F_20,4_ = 1.086, *p* = 0.404) did not. In the phylogenetic ANOVA with combined factors of burrowing + locomotion + diet on the combined limb data set, none of the factors were significant (F_20,2_ = 3.265, *p* = 0.167; F_20,2_ = 1.502, *p* = 0.314; F_20,1_ = 0.877, *p* = 0.388; respectively). Similarly, in the factorial phylogenetic ANOVA between burrowing and locomotion, neither the factors (F_20,2_ = 3.161, *p* = 0.186 and F_20,2_ = 1.454, *p* = 0.344, respectively) nor the interaction (F_20,1_ = 0.371, *p* = 0.818) were significant. The factorial ANOVA between burrowing and diet, and the factorial phylogenetic ANOVA between diet and locomotion were also not significant. These results were slightly different when looking at forelimbs and hindlimbs data sets separately. On the forelimbs GM data set (RU + H), burrowing (F_20,2_ = 3.8343, *p* = 0.003) and diet (F_20,1_ = 4.383, *p* = 0.002) were significant, whereas locomotor mode (F_20,2_ = 1.310, *p* = 0.196) and biome were not significant (F_20,4_ = 0.6608, *p* = 0.271). In the phylogenetic ANOVA with the three factors (diet + burrowing + locomotion), only burrowing was significant (F_20,2_ = 5.543, *p* = 0.012). In the factorial ANOVA between burrowing and locomotion, only burrowing (F_20,2_ = 4.453, *p* = 0.021) was significant. In the factorial ANOVA between diet and burrowing, both factors were significant (F_20,1_ = 5.534, *p* = 0.004 and F_20,2_ = 3.448, *p* = 0.018, respectively) but the interaction was not (F_20,1_ = 1.095, *p* = 0.292). Finally, in the factorial ANOVA between diet and locomotion, only diet (F_20,1_ = 4.202, *p* = 0.012) was significant. Finally, on the hindlimbs GM data set, burrowing (F_20,2_ = 5.177, *p* = 0.013) was also significant, whereas locomotor mode (F_20,2_ = 1.316, *p* = 0.251), diet (F_20,1_ = 0.881, *p* = 0.252), and biome (F_20,4_ = 0.708, *p* = 0.687) were not. In the phylogenetic ANOVA with the three factors (burrowing + locomotion + diet) on the hindlimb GM data set, only burrowing was significant (F_20,2_ = 6.361, *p* = 0.038). In the factorial ANOVA between burrowing and locomotion, only burrowing was significant (F_20,2_ = 7.084, *p* = 0.027). None of the factors nor the interactions were significant in the factorial ANOVAs between burrowing and diet, and diet and locomotion.

## Discussion

We evaluated morphological differences in skulls and limb bones on representative species from all 21 genera of Australian myobatrachid frogs, using a 3D geometric morphometric approach on multiple structures. With this method we were able to focus on the tempo and mode of morphological evolution in this old Gondwanan radiation by asking three main questions: (1) whether morphological evolutionary patterns are similar for different structures, (2) if the amount and direction of morphological change differs for each structure and clade, and (3) if morphological evolution is correlated to functional traits such as locomotion, burrowing, or diet. We found that both head and limbs followed a complex evolutionary pattern typical of adaptive radiation, followed by a Brownian Motion process. Nevertheless, there was a low level of morphological integration between the skull and the limbs and there were significant differences in the mode of morphological evolution between the head and limbs. Skull morphology was phylogenetically conserved and correlated to diet, whereas limb morphology was more labile within clades and appeared to be shaped by diet, burrowing behavior and locomotion. Morphological differences among different limb modules suggest co-variation and strong morphological integration due to selection and functional constraints imposed by burrowing and locomotion. Our results illustrate how morphological diversification in animal clades can follow complex processes, entailing selective pressures from the environment as well as multiple trait covariance with varying degrees of independence across different structures. We discuss each of these topics in turn, and suggest that accurately quantifying shape diversity across multiple structures is crucial in order to understand complex evolutionary processes.

We showed that different phylogenetic clades were separated in skull morphospace, suggesting an early diversification of head shape in myobatrachid frogs, which was supported by an Early Burst model of phenotypic evolution followed by a Brownian Motion process. The majority of skull differences were correlated with fenestration: the subfamily Limnodynastinae displayed bigger and rounder orbits, and robust sphenethmoids and parasphenoids, while species from the Myobatrachinae subfamily generally showed more elongate orbits and larger antorbital fenestrae. The two other major clades, *Rheobatrachus* (comprising the two extinct gastric-brooding frog species) and *Mixophyes* spp. (8 extant species of barred frogs), displayed skull shapes that were intermediate to Limnodynastinae and Myobatrachinae. This pattern of early morphological diversification suggests that occupancy of new morphospace regions by ancestral lineages of myobatrachids could have been associated with major ecological niche filling processes that are typical of diversifying lineages [11, 74, 75]. Analogous with skull shape diversification, morphological evolution in the limbs was best explained by a complex model of an Early Burst process, followed by Brownian Motion. This was unexpected, as myobatrachid frogs are an old Gondwanan adaptive radiation, displaying an exceptionally high degree of ecological, behavioural and morphological diversity across the whole Australian continent. However, phylogenetic non-independence of highly dimensional data, such as 3D GM data, involves some potential pitfalls when inferring complex evolutionary processes, as exposed by Uyeda et al. [76]. For example, Early Burst processes could arise as an artefact from discretising highly dimensional data sets and examining a relatively small sample from multivariate patterns. Thus, caution should be used when inferring evolutionary processes on high effective dimensionality, and our initial results on myobatrachid clades would most likely benefit from a more extensive sampling within genera.

Myobatrachid frogs have experienced several major geological and climatic processes that would have affected diversification and ecological and morphological evolution [41]. Our PCA analyses of the humerus, radioulna, and whole forelimb (H + RU), distributed forward burrowers and most walkers in one broad region of the morphospace, with jumpers/swimmers, backward and non-burrowers, and one walker grouped together on the opposite side of the morphospace. The most extreme forelimb shape was exhibited by forward burrowers that displayed a stronger and more robust humerus, with larger lateral epicondyles, extremely robust radioulnas with large olecranons and a conspicuous longitudinal groove between the radius and ulna. In contrast, good jumpers or swimmer species from wet environments, such as *Lechriodus* sp. and the extinct *Rheobatrachus* spp., generally displayed slender forelimb bones with less pronounced arching, and a faint longitudinal groove in the radioulna. For the hindlimbs (F + TF), shape diversity was mostly strongly correlated with burrowing behavior (both forward burrowers and clades in which all species are backward burrowers). Both the femur and tibiofibula were shorter and thicker in burrowing species, and displayed pronounced arching and tuberosities (such as the third trochanter) to facilitate muscle attachments.

We found that diet, burrowing, and locomotion played an important role in shaping morphological diversification of myobatrachid frogs. Skull morphology was associated with diet, with ant and termite specialist feeders displaying shorter snouts than generalist species. Several other taxa, including lizards [77], crocodiles [78], mammals [22, 23], and turtles [79] also show clear associations between diet and skull shape. We also found a strong correlation between skull shape and functional traits, such as burrowing and locomotion. That is not an unexpected result, while ecotype, habitat, and other environmental and climate has been found to not have an impact in skull shape diversification in some clades [80], it can also greatly influence head shape in some clades [79, 81, 82], it can also have no impact in others.

Shape diversification of limb bones was not as strongly correlated with phylogenetic history, and instead, diet, locomotor type and burrowing behavior seemed to be important contributors to the morphological variation observed among species. Even though each limb bone displayed slight differences in their shape diversification and its correlation with different ecological variables, they did not differ substantially overall, probably due to their high morphological integration. There was, however, certain degree of independence between fore and hindlimbs, mostly due to functional requirements. Both fore and hindlimbs were correlated to burrowing behavior, but only forelimbs were associated with dietary requirements. The fact that locomotion was strongly correlated with the overall limb shape but not each module or fore and hind limbs independently is not surprising, as fore-to-hind-limb ratios have been proved to be important in explaining locomotor abilities in different frog clades [37]. While most frogs and toads explosive jumping energy is produced by the hind-limbs, they land on their adducted forelimbs, which play a critical role in locomotion by determining the landing and stabilizing actions that enable the next jumping phase [83–85]. Thus, our results suggest that limb shape might have evolved as a response to locomotion constraints imposed by different structural habitats, which would constrain the locomotor modes. This concurs with results found in other amphibian clades, where variation in habitat use and locomotor behaviour seem to correlate with particular ratios between fore and hindlimb lengths [26, 27, 37]. The same trend also is noticeable in other vertebrate groups, such as phrynosomatid lizards [86], anoles [87], sauropods [88], and carnivorous mammals [89, 90], in which ecotype or locomotor type is correlated with limb morphology or distinct proportions between fore and hindlimbs.

The study of locomotion is fundamental to understanding animal biology, as it links morphology with the use of different environments through navigation, feeding, and escape from predators [91]. In addition, factors such as ecology and some less-conspicuous behavioural aspects could also contribute to morphological evolution, making inferences about evolutionary history difficult [24]. Because multiple variables can create selective pressures in different directions on phenotypic traits, their interactions could potentially lead to trade-offs. For example, morphological optimisation for burrowing creates opposing pressures from optimisation for jumping, due to discordances in functional morphological requirements for each behaviour [38]. Although myobatrachid frogs generally display phylogenetic conservatism in morphology, burrowing behavior and other ecological correlates still appear to have a strong effect on limb shape. Morphological adaptations in forward burrowers are primarily associated with forelimb bones in both amphibians and other fossorial vertebrates [92]. Similarly, despite not being found in any other vertebrate, backward burrowing represents 95% of all burrowing types in anurans [34]. The evolution of both forward and backward burrowing likely led to reduced length and increased robustness of fore and hindlimbs respectively, which would almost certainly have resulted in reduced locomotor abilities [30, 38]. This trend towards shorter and more robust limbs in burrowing anurans is likely also a beneficial adaptation to arid environments. Amphibians have adapted to a wide range of extreme climatic conditions, despite experiencing more constraints than any other terrestrial vertebrate due to rapid evaporative water loss through their permeable skin [34]. By reducing limb length, total surface area of the body can also be reduced and with it, evaporative water loss.

Despite high overall morphological disparity among different myobatrachid genera [45], some structures (e.g. limbs) displayed morphological integration and co-variation leading to convergent phenotypes, while other structures (e.g. skulls) followed semi-independent evolutionary processes. Despite the low integration between skull and all post-cranial modules, hind- and forelimbs were more tightly correlated to the skull when assessed independently, especially the hindlimbs. These results could be due to a certain degree of integration between head and postcranial modules, which could follow different directions in the morphospace for each limbs module, resulting in semi-independent pattern of morphological evolution of the head versus the rest of the body. Our results, therefore, support the modularity or semi-independent hypothesis when looking at morphological evolution between skulls and limbs, but favours the morphological integration hypothesis for shape diversification within different limb modules, or the different substructures within the skull. Thus, while high evolutionary lability experienced by limbs is a result of selective pressures from the environment, skulls instead display relatively high phylogenetic conservatism. This suggests that morphological diversification might have occurred rapidly quite early in the myobatrachid frog radiation, followed by a decrease in shape disparity, which is conspicuous through the different areas of skull morphospace. Head shape in anurans appears to have undergone extreme morphological change very early in the evolutionary history of modern amphibians, which is especially conspicuous through a substantial widening of the skull and orbits, and enlargement of fenestrae [76, 93]. Moreover, strong phylogenetic structure on skull shape is not unusual among other amphibian groups older than 50 MY (e.g. caecilians [75]), in contrast to younger vertebrate radiations that typically display greater morphological disparity, with weaker phylogenetic signal [94].

Phylogenetic conservatism and morphological diversification in functional traits can provide insight into evolutionary processes [24], but the interplay between different potential drivers of adaptation can blur the link between form and function. For example, limb morphology might appear strongly correlated with locomotion type, but habitat use or burrowing behaviour might be equally important correlates. In this way adaptive traits often cannot (and should not) be explained by just one adaptive process. Morphological integration or modularity also can affect the accuracy of evolutionary inferences on adaptation to certain ecological, locomotor or behavioural factors [95]. Furthermore, rates of phenotypic evolution can be correlated with species diversification rates within clades, as morphological traits typically have slower evolutionary rates than other traits such as behaviour [67, 96]. Moreover, closely related clades might display unequal magnitudes of morphological change, thus hindering or boosting apparent morphological diversification, especially early in their evolutionary history [11].

## Conclusions

Our study is the first to accurately identify evolutionary processes that drive morphological diversity in the context of modularity and morphological integration of several structures in an old adaptive radiation and across a whole continent. Our results highlight how form is usually tightly linked to function, and that different structures can evolve semi-independently, while in other modules morphological evolution is tightly coupled. There was strong morphological co-variation among different modules in the limbs due to strong selective pressures from the environment and functional trade-offs (e.g. burrowing and locomotion), whereas skull shape was correlated to diet, and yield a pattern of very early morphological diversification followed by strong phylogenetic conservatism. Our results also show that even when different structures evolve following the same evolutionary models, patterns of morphological diversification can be drastically different. The complex interplay between selective pressures and different levels of co-varying morphological evolution makes it harder to accurately identify processes that drive clade diversification and infer their evolutionary history. Thus, we highlight the importance of accurately assessing morphological evolution in multiple structures in order to properly understand complex evolutionary processes that generate the phenotypic diversity we see today.

## Additional files

Additional file 1: **Table S1.** Summary of several ecological and behavioural traits of the myobatrachid frogs studied here, used in posterior analyses: burrowing behaviour, locomotor mode, habitat type or ecoregion, and diet type. **Table S2.** Summary of the different landmarks used for each module (m1, m2 or m3) in all five models of modular partitions (bimodular and trimodular) within the skull that correspond to the models displayed on Additional file 5: Figure S2. **Table S3.** Principal Component Analyses of shape variation for different sets of Procrustes-aligned species means, using *geomorph*. **Table S4.** Summary of phylogenetic signal tests, using *geomorph* (Adams & Otarola-Castillo, 2013). K 95% confidence interval for values expected under a Brownian Motion model of trait evolution = [0.799, 1.318]. **Table S5.** Summary statistics for the fit of models of phenotypic evolution in the first five principal components of the Skull shape dataset and the limbs shape dataset (all four limb bones together): maximum likelihood estimate (ln L), sample-size corrected Akaike’s Information Criterion (AICc), and Delta AICc (ΔAICc, difference between a model and the model with the lowest AICc). We tested the fit of the following evolutionary models: BM = Brownian Motion, EB = Early Burst, white = nonphylogenetic, OU = Ornstein-Uhlenbeck, OU2_diet = Ornstein-Uhlenbeck with two optima based on diet, OU3_loc = OU with three optima based on locomotion, and OU3_burr = OU with three optima based on burrowing behaviour. Analyses were performed in R using *geiger* [68] and *ouch* [69]. **Table S6.** Results from the *integration.test* function in geomorph (Adams & Otrola-Castillo, 2013) in order to quantify the degree of modularity between the two or three modules in each modular configuration (a-e), using the landmark coordinate data. **Appendix S1.** Species and specimen codes for all the individuals used in this study, by museums. (PDF 171 kb)

Additional file 2: Video displaying the 42 landmarks used in the GM analyses of the skull. (PDF 25415 kb)

Additional file 3: Video displaying both the landmarks and semi-landmarks used in the GM analyses of the four limb bones: radioulna, humerus, tibiofibular and femur. (PDF 6874 kb)

Additional file 4: Figure S1. (a) Phylomorphospace of PCA values on shape variation of radioulna (RU); (b) Phylomorphospace of PCA values on shape variation of tibiofibula (TF); (c) Phylomorphospace of PCA values on shape variation of humerus (H); (d) Phylomorphospace of PCA values on shape variation of femur (F); (e) Phylomorphospace of PCA values on fore-limb shape variation (RU + H); (f) Phylomorphospace of PCA values on hind-limb shape variation (TF + F). (PDF 332 kb)

Additional file 5: Figure S2. Modular configurations modeled for the skull with two or three different partitions, based on different evolutionary hypothesis based on biological relevant regions. The different colours depict different modules. (a) The first module (green) includes the tip of the snout and the olfactory area (premaxilla, maxilla, and nasal), as it captures a lot of morphological variation among frog species, whereas the second module (blue) includes the rest of the skull; (b) this configuration captures skull depth – the first module includes the dorsal region of the skull, and the second module captures morphological information from the ventral region; (c) this tripartite model splits the skull in three modules: snout (green), squamosal (orange, which is part of the suspensory apparatus), and the rest of the skull (blue); (d) The first module depicts the snout (green), the second includes the medial part of the skull (orange), and the third module includes the most posterior region of the skull (blue); (e) this tripartite configuration includes a first module (green) with the snout morphology, a second module (orange) that encompasses the brain region (from the sphenethemoid to the exoccipital and foramen magnum, including the frontoparietal), and a third module (blue) for the rest of the skull. (PDF 275 kb)
