# Supplementary material for: Phylogenetic conservatism in skulls and evolutionary lability in limbs – morphological evolution across an ancient frog radiation is shaped by diet, locomotion and burrowing
Source: BMC Evol Biol. 2017 Jul 10;17:165. doi: 10.1186/s12862-017-0993-0 (PMC5504843; doi:10.1186/s12862-017-0993-0)
Supplement: Supplementary file 1 — Table S1. Summary of several ecological and behavioural traits of the myobatrachid frogs studied here, used in posterior analyses: burrowing behaviour, locomotor mode, habitat type or ecoregion, and diet type. Table S2. Summary of the different landmarks used for each module (m1, m2 or m3) in all five models of modular partitions (bimodular and trimodular) within the skull that correspond to the models displayed on Additional file 5: Figure S2. Table S3. Principal Component Analyses of shape variation for different sets of Procrustes-aligned species means, using geomorph. Table S4. Summary of phylogenetic signal tests, using geomorph (Adams & Otarola-Castillo, 2013). K 95% confidence interval for values expected under a Brownian Motion model of trait evolution = [0.799, 1.318]. Table S5. Summary statistics for the fit of models of phenotypic evolution in the first five principal components of the Skull shape dataset and the limbs shape dataset (all four limb bones together): maximum likelihood estimate (ln L), sample-size corrected Akaike’s Information Criterion (AICc), and Delta AICc (ΔAICc, difference between a model and the model with the lowest AICc). We tested the fit of the following evolutionary models: BM = Brownian Motion, EB = Early Burst, white = nonphylogenetic, OU = Ornstein-Uhlenbeck, OU2_diet = Ornstein-Uhlenbeck with two optima based on diet, OU3_loc = OU with three optima based on locomotion, and OU3_burr = OU with three optima based on burrowing behaviour. Analyses were performed in R using geiger [68] and ouch [69]. Table S6. Results from the integration.test function in geomorph (Adams & Otrola-Castillo, 2013) in order to quantify the degree of modularity between the two or three modules in each modular configuration (a-e), using the landmark coordinate data. Appendix S1. Species and specimen codes for all the individuals used in this study, by museums. (PDF 171 kb) [file 12862_2017_993_MOESM1_ESM.pdf]

Table S1. Summary of several ecological and behavioural traits of the myobatrachid frogs studied here, used in posterior analyses: burrowing behaviour, locomotor mode, habitat type or ecoregion, and diet type.

Species	Burrowing	Locomotion	Ecoregion	Diet
**Adelotus**	non-burrower	jumper/swimmer	temperate & tropical	generalist
**Arenophryne**	forward	walker	mediterranean	specialist
**Assa**	non-burrower	walker	temperate	generalist
**Crinia**	non-burrower	jumper/swimmer	all ecoregions	generalist
**Geocrinia**	non-burrower	hopper	temperate & mediterranean	generalist
**Heleioporus**	backward	hopper	temperate & mediterranean	generalist
**Lechriodus**	non-burrower	jumper/swimmer	temperate	generalist
**Limnodynastes**	backward	jumper/swimmer	all ecoregions	generalist
**Metacrinia**	non-burrower	walker	mediterranean	specialist
**Mixophyes**	non-burrower	jumper/swimmer	temperate & tropical	generalist
**Myobatrachus**	forward	walker	mediterranean	specialist
**Neobatrachus**	backward	hopper	all ecoregions	generalist
**Notaden**	backward	hopper	all ecoregions	generalist
**Paracrinia**	non-burrower	jumper/swimmer	temperate	generalist
**Philoria**	non-burrower	hopper	temperate	generalist
**Platyplectrum**	backward	hopper	temperate & tropical	generalist
**Pseudophryne**	backward	hopper	temperate & mediterranean	generalist
**Rheobatrachus**	non-burrower	jumper/swimmer	temperate & tropical	generalist
**Spicospina**	non-burrower	walker	mediterranean	generalist
**Taudactylus**	non-burrower	jumper/swimmer	temperate & tropical	generalist
**Uperoleia**	backward	hopper	all ecoregions	generalist

Table S2: Summary of the different landmarks used for each module (m1, m2 or m3) in all five models of modular partitions (bimodular and trimodular) within the skull that correspond to the models displayed on Fig. S2.

Landmark	a	b	c	d	e
**1**	m1	m1	m1	m1	m1
**2**	m1	m1	m1	m1	m1
**3**	m1	m1	m1	m1	m1
**4**	m1	m1	m1	m1	m1
**5**	m1	m1	m1	m1	m1
**6**	m1	m1	m1	m1	m1
**7**	m2	m1	m2	m2	m2
**8**	m2	m1	m2	m2	m2
**9**	m2	m1	m2	m2	m2
**10**	m2	m1	m2	m2	m2
**11**	m2	m2	m2	m2	m3
**12**	m2	m2	m2	m2	m3
**13**	m2	m2	m2	m2	m3
**14**	m2	m2	m2	m2	m3
**15**	m2	m2	m2	m2	m3
**16**	m2	m2	m2	m2	m3
**17**	m2	m1	m2	m3	m3
**18**	m2	m1	m2	m3	m3
**19**	m2	m1	m2	m3	m2
**20**	m2	m1	m2	m3	m2
**21**	m2	m1	m2	m3	m2
**22**	m2	m1	m2	m3	m2
**23**	m2	m1	m2	m3	m2
**24**	m2	m1	m2	m3	m2
**25**	m2	m1	m2	m3	m2
**26**	m2	m1	m2	m3	m2
**27**	m2	m1	m2	m2	m2
**28**	m2	m1	m2	m2	m2
**29**	m2	m1	m2	m3	m3
**30**	m2	m1	m2	m3	m3
**31**	m2	m2	m2	m3	m3
**32**	m2	m2	m2	m3	m3
**33**	m2	m2	m2	m3	m3
**34**	m2	m2	m2	m3	m3
**35**	m1	m2	m1	m1	m1
**36**	m1	m2	m1	m1	m1
**37**	m2	m2	m3	m3	m3
**38**	m2	m2	m3	m3	m3
**39**	m2	m2	m3	m3	m3
**40**	m2	m2	m3	m3	m3
**41**	m2	m2	m3	m3	m3
**42**	m2	m2	m3	m3	m3

Table S3. Principal Component Analyses of shape variation for different sets of Procrustes-aligned species means, using *geomorph*.

PCA data set	AXIS 1		AXIS 2		AXIS 3		AXIS 4		AXIS 5	
	SD	Variance (%)	SD	Variance (%)	SD	Variance (%)	SD	Variance (%)	SD	Variance (%)
Skull	0.108	41.580	0.074	19.720	0.054	10.310	0.043	6.550	0.034	4.070
Radioulna (RU)	0.066	57.990	0.023	7.230	0.020	5.370	0.018	4.470	0.015	3.000
Humerus (H)	0.073	39.380	0.056	23.140	0.036	6.810	0.030	4.580	0.025	2.880
Forearms (RU+H)	0.052	47.220	0.027	12.240	0.025	10.610	0.020	6.830	0.018	5.700
Tibiofibula (TF)	0.134	62.010	0.063	13.580	0.052	9.190	0.041	5.720	0.035	4.320
Femur (F)	0.119	81.230	0.041	9.880	0.027	4.050	0.016	1.460	0.012	0.830
Hindlimbs (TF+F)	0.060	75.340	0.022	9.770	0.017	5.900	0.010	2.200	0.009	1.510
Limbs (RU+H+TF+F)	0.059	45.460	0.053	37.470	0.018	4.280	0.013	2.210	0.012	1.770

Table S4. Summary of phylogenetic signal tests, using *geomorph* (Adams & Otarola-Castillo, 2013). K 95% confidence interval for values expected under a Brownian Motion model of trait evolution = [0.799, 1.318].

**Variable**	$\mathbf{K}_{\mathrm{mult}}$	**p**
Skull	1.0724	**0.001**
Radioulna (RU)	0.9196	0.3382
Humerus (H)	0.9314	0.2198
Tibiofibula (TF)	1.1315	**0.0119**
Femur (F)	1.0853	**0.0171**
Arm (RU + H)	0.9056	0.3458
Leg (TF + F)	0.8654	0.5798
Limbs (RU + H + TF + F)	1.0746	**0.001**
Body (Skull + RU + H + TF + F)	0.9065	0.2937

Table S5: Summary statistics for the fit of models of phenotypic evolution in the first five principal components of the Skull shape dataset and the limbs shape dataset (all four limb bones together): maximum likelihood estimate (ln L), sample-size corrected Akaike's Information Criterion (AICc), and Delta AICc (ΔAICc, difference between a model and the model with the lowest AICc). We tested the fit of the following evolutionary models: BM = Brownian Motion, EB = Early Burst, white = non-phylogenetic, OU = Ornstein-Uhlenbeck, OU2_diet = Ornstein-Uhlenbeck with two optima based on diet, OU3_loc = OU with three optima based on locomotion, and OU3_burr = OU with three optima based on burrowing behaviour. Analyses were performed in R using *geiger* (XXX) and *ouch* (XXX).

Variable	BM	EB	white	OU	OU2_diet	OU3_loc	OU3_burr
**SKULL PC 1**							
ln L	18.655	19.330	17.458	18.655	21.321	25.638	24.385
AICc	-32.643	-31.247	-30.250	-29.898	-32.143	-37.276	-34.770
ΔAICc	4.63378	6.029	7.02655	7.37888	5.134	**0**	2.506
**SKULL PC 2**							
ln L	30.389	32.031	25.289	30.389	26.296	30.290	31.636
AICc	-56.112	-56.650	-45.912	-53.367	-42.093	-46.581	-49.272
ΔAICc	0.538	**0**	10.739	3.283	14.558	10.070	7.378
**SKULL PC 3**							
ln L	32.908	33.473	32.097	32.908	34.011	33.454	33.658
AICc	-61.149	-59.534	-59.528	-58.404	-57.523	-52.909	-53.316
ΔAICc	**0**	1.615	1.622	2.745	3.627	8.241	7.834
**SKULL PC 4**							
ln L	36.859	36.859	36.861	37.077	38.981	37.914	37.573
AICc	-69.051	-66.306	-69.056	-66.742	-67.463	-61.828	-61.146
ΔAICc	0.005	2.750	**0**	2.314	1.593	7.228	7.910
**SKULL PC 5**							
ln L	38.553	38.553	41.869	41.615	42.736	41.873	42.012
AICc	-72.439	-69.694	-79.071	-75.817	-74.972	-69.746	-70.025
ΔAICc	6.632	9.377	**0**	3.254	4.100	9.325	9.047
**LIMBS PC 1**							
ln L	33.529	38.094	30.243	33.529	32.640	33.739	33.996
AICc	-62.391	-68.775	-55.820	-59.646	-54.779	-53.477	-53.992
ΔAICc	6.384	**0**	12.956	9.129	13.996	15.298	14.784
**LIMBS PC 2**							
ln L	31.751	31.751	32.274	32.453	37.300	37.873	38.322
AICc	-58.836	-56.091	-59.881	-57.494	-64.100	-61.747	-62.643
ΔAICc	5.264	8.009	4.219	6.606	**0**	2.353	1.456
**LIMBS PC 3**							
ln L	52.901	52.901	55.050	54.920	56.901	56.120	59.735
AICc	-101.136	-98.390	-105.432	-102.428	-103.303	-98.241	-105.471
ΔAICc	4.335	7.080	0.038	3.043	2.168	7.230	**0**
**LIMBS PC 4**							
ln L	60.085	60.085	62.018	61.866	62.266	62.602	62.264
AICc	-115.503	-112.758	-119.370	-116.321	-114.032	-111.204	-110.529
ΔAICc	3.867	6.612	**0**	3.049	5.338	8.166	8.841
**LIMBS PC 5**							
ln L	61.945	61.945	64.344	64.163	64.418	65.577	64.801
AICc	-119.223	-116.478	-124.022	-120.914	-118.336	-117.154	-115.601
ΔAICc	4.798	7.543	**0**	3.108	5.686	6.868	8.421

Table S6. Results from the *integration.test* function in geomorph (Adams & Otrola-Castillo, 2013) in order to quantify the degree of modularity between the two or three modules in each modular configuration (a-e), using the landmark coordinate data. All tests were based on 10,000 random permutations, with r-PLS being the estimate of morphological integration between two or more partitions, and p indicating the significance of the test.

**Modular configuration**	**r-PLS**	**p**
Bimodular 1 (a)	0.947	0.001
Bimodular 2 (b)	0.972	0.001
Trimodular 1 (c)	0.942	0.008
Trimodular 2 (d)	0.934	0.032
Trimodular 3 (e)	0.912	0.045

Appendix S1

Species and specimen codes for all the individuals used in this study, by museums

AM: *Adelotus brevis* (R128155, R128156, R128157), *Assa darlingtoni* (R80514, R80521), *Crinia signifera* (R172161, R172172), *Geocrinia leai* (R39281, R39295), *Heleioporus albopunctatus* (R39180, R39376), *Lechriodus fletcheri* (R104371, R132817, R138937), *Limnodynastes salmini* (R35173, R36157), *Metacrinia nichollsi* (R55986, R98291), Mixophyes fasciolatus (R139040, R139300), *Neobatrachus sudelli* (R51248, R51251), *Notaden bennettii* (R156793, R165793), *Paracrinia haswelli* (R111264, R162984), *Philoria sphagnicolus* (R130933, R139084), *Platyplectrum ornatum* (R138263, R143309), *Pseudophryne coriacea* (R161034, R167900), *Taudactylus eungellensis* (R47789, R47799), *Uperoleia laevigata* (R36100, R36838). **WAM:** *Arenophryne xiphorhyncha* (R126259, R126272), *Metacrinia nicholsii* (R146301, R146078), *Myobatrachus gouldii* (R149640, R146210), *Spicospina flammocaerulea* (R112150).
