# Supplementary figures and images for: Phylogenetic conservatism in skulls and evolutionary lability in limbs – morphological evolution across an ancient frog radiation is shaped by diet, locomotion and burrowing
Source: BMC Evol Biol. 2017 Jul 10;17:165. doi: 10.1186/s12862-017-0993-0 (PMC5504843; doi:10.1186/s12862-017-0993-0)

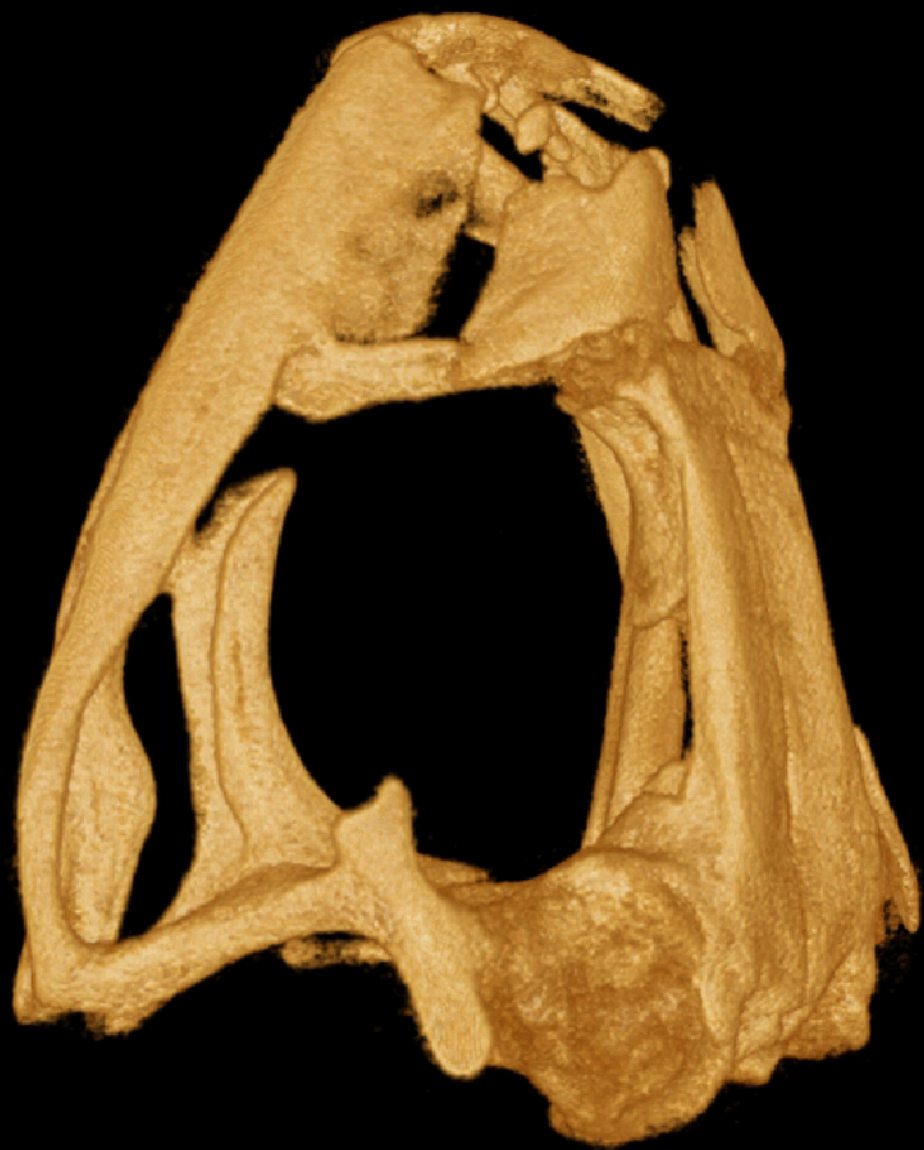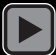

Supplement: Supplementary file 2 — Video displaying the 42 landmarks used in the GM analyses of the skull. (PDF 25415 kb) [file 12862_2017_993_MOESM2_ESM.pdf]

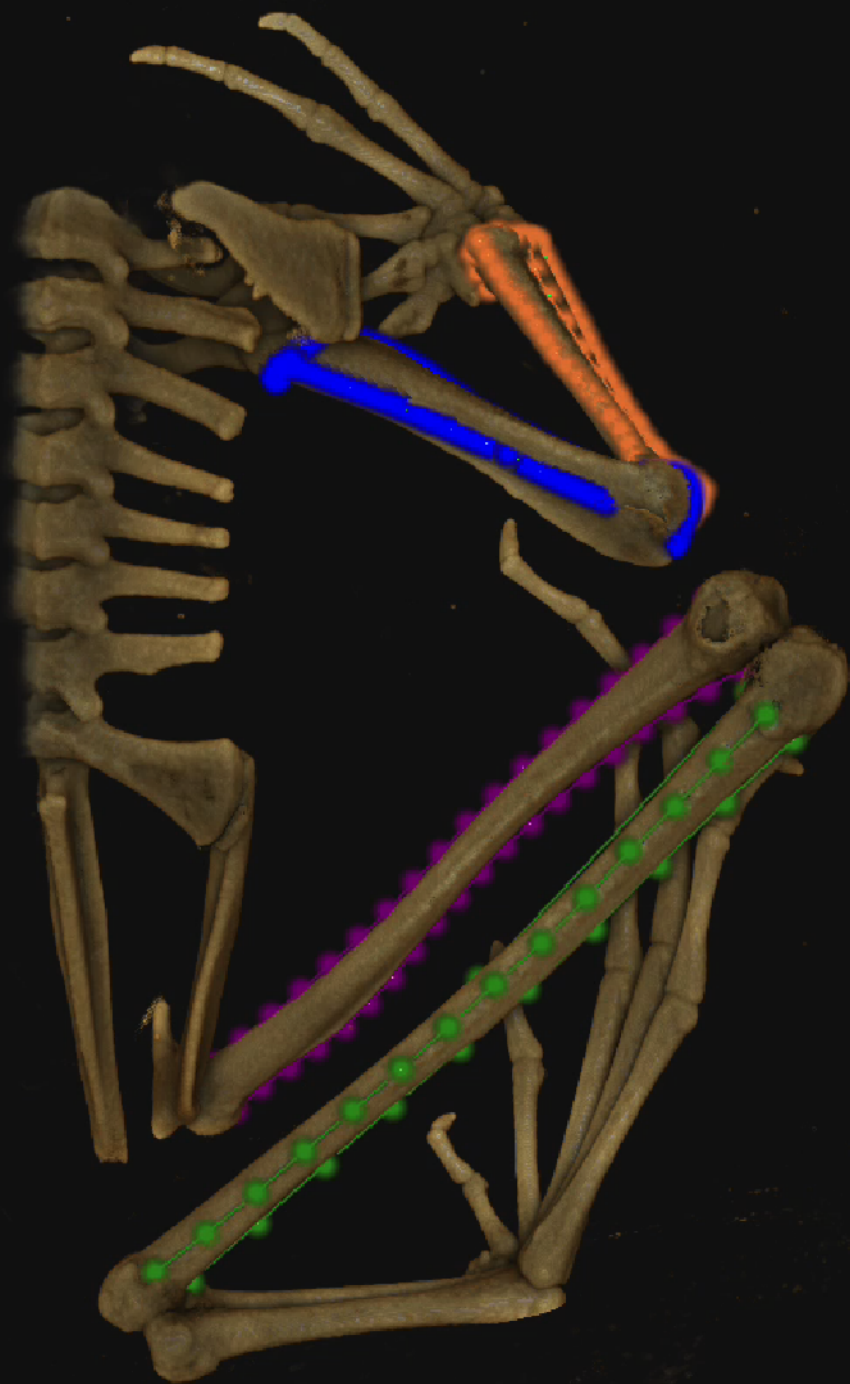

Supplement: Supplementary file 3 — Video displaying both the landmarks and semi-landmarks used in the GM analyses of the four limb bones: radioulna, humerus, tibiofibular and femur. (PDF 6874 kb) [file 12862_2017_993_MOESM3_ESM.pdf]

(a)

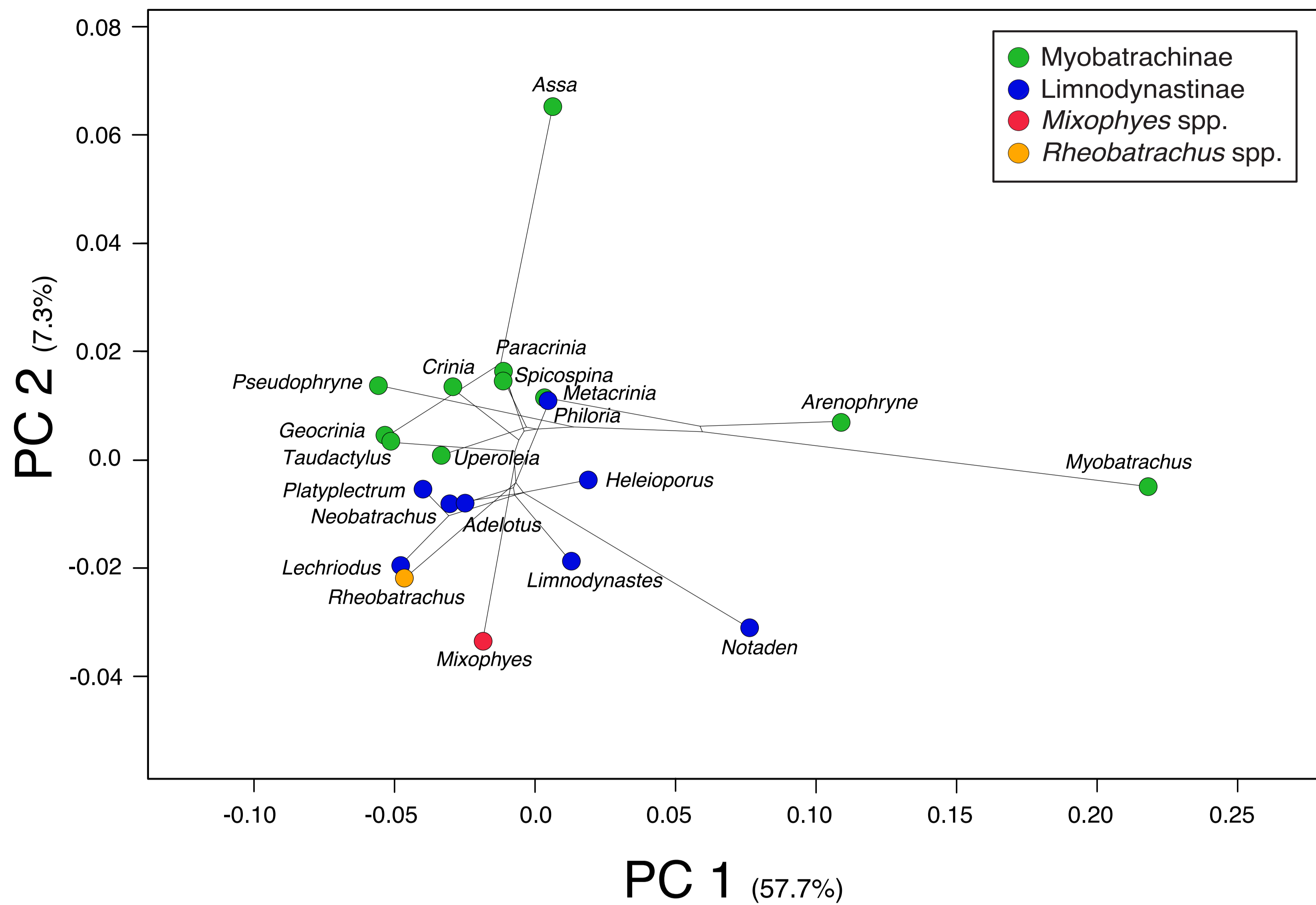

(b)

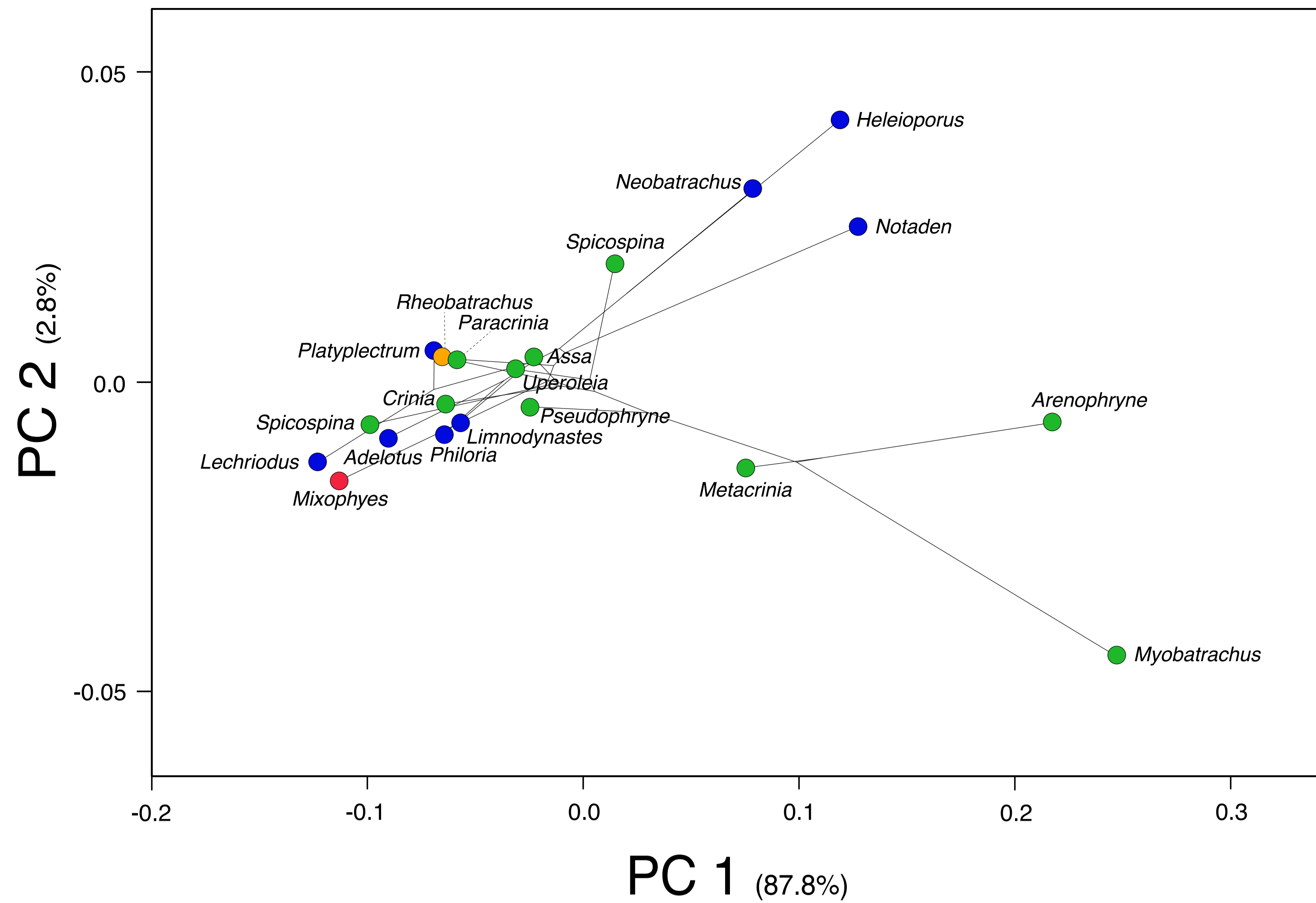

(c)

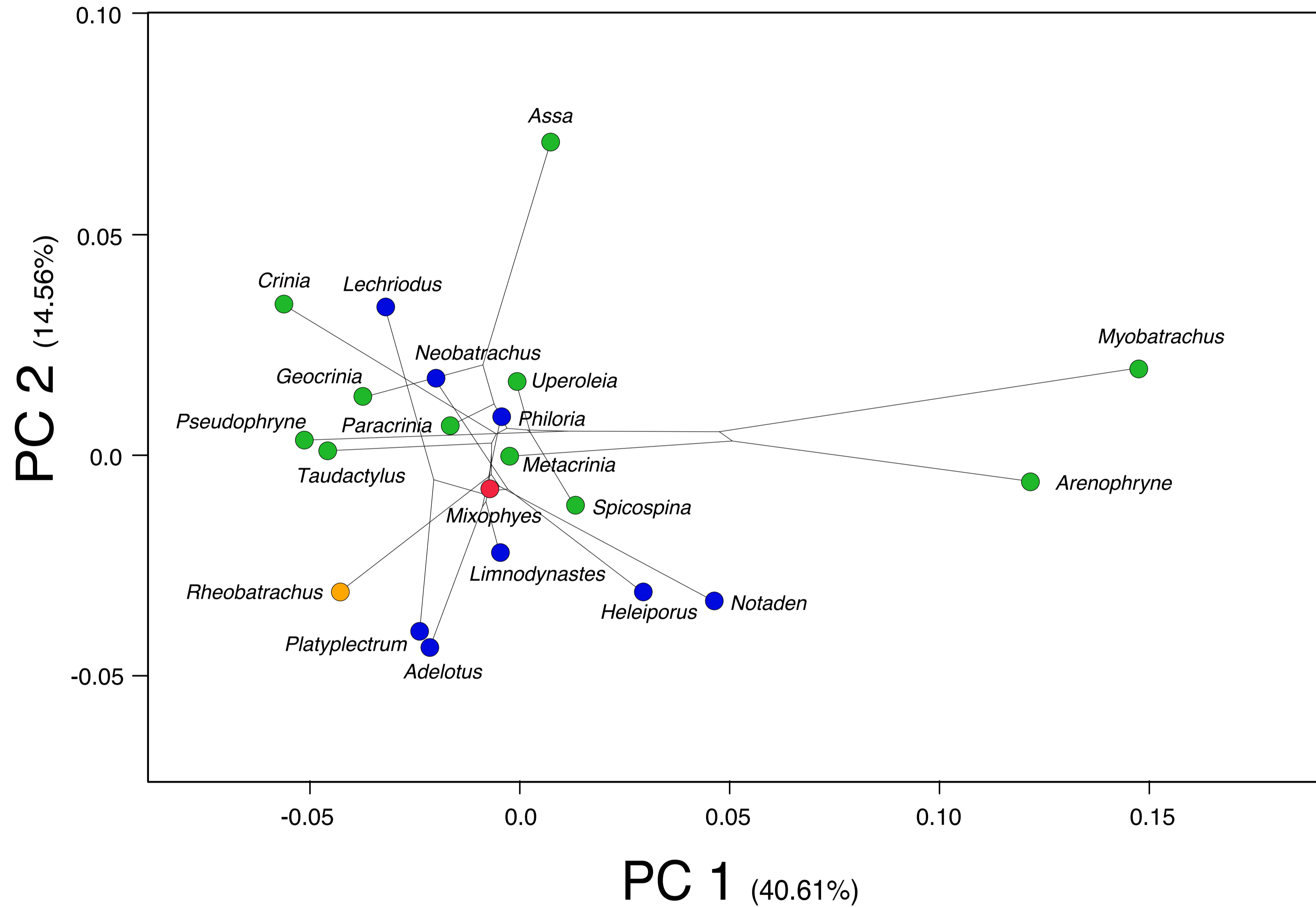

(d)

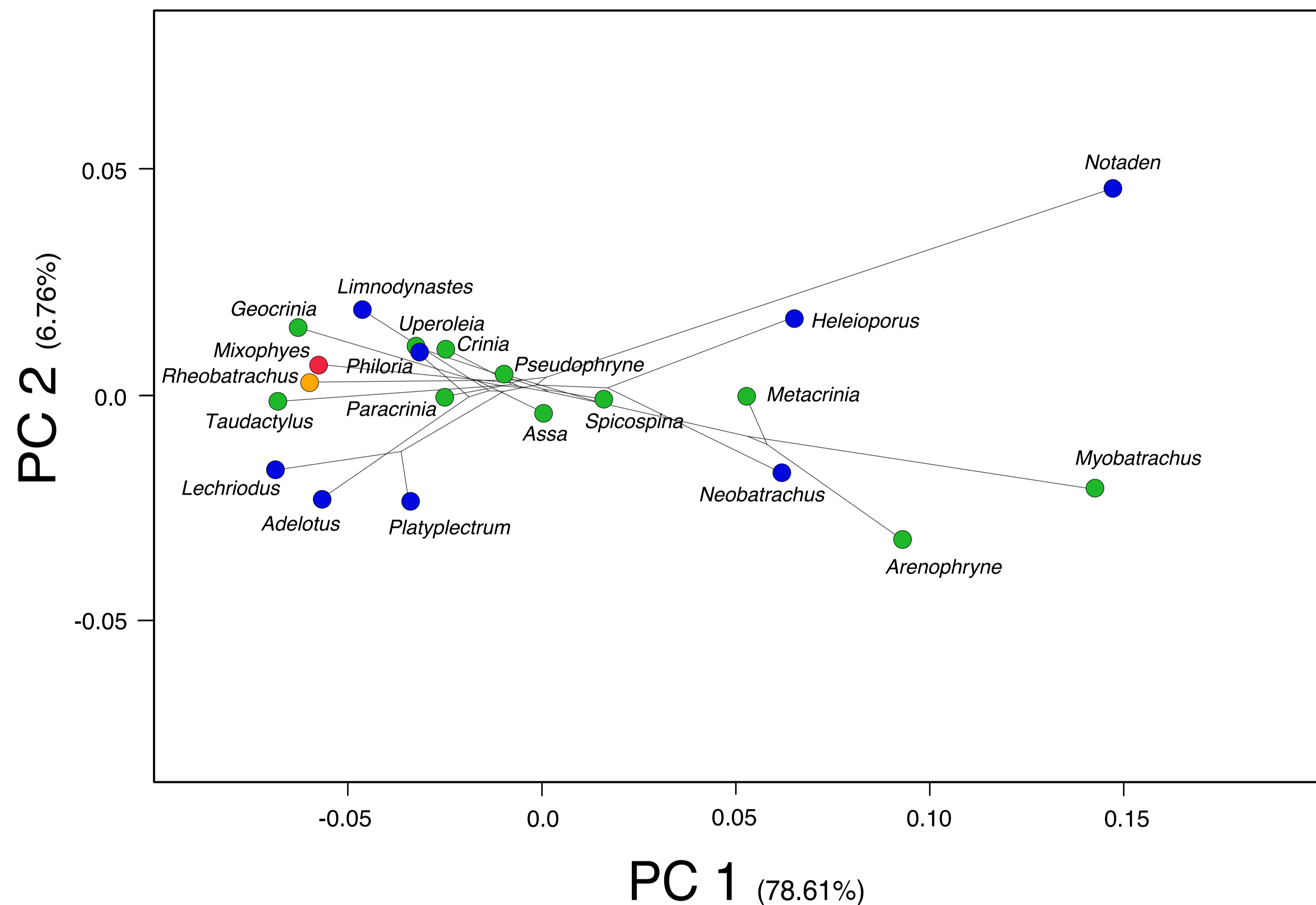

(e)

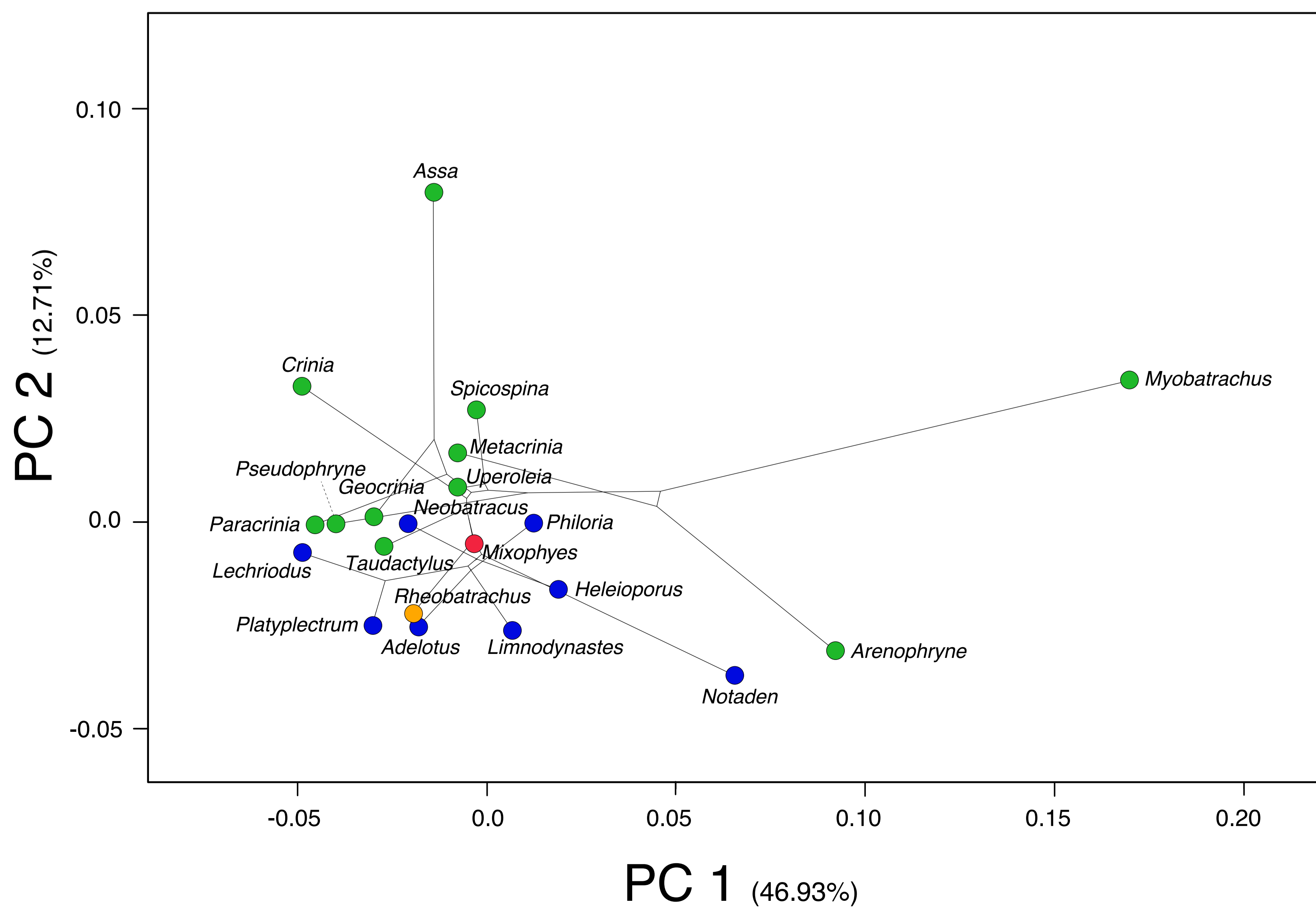

(f)

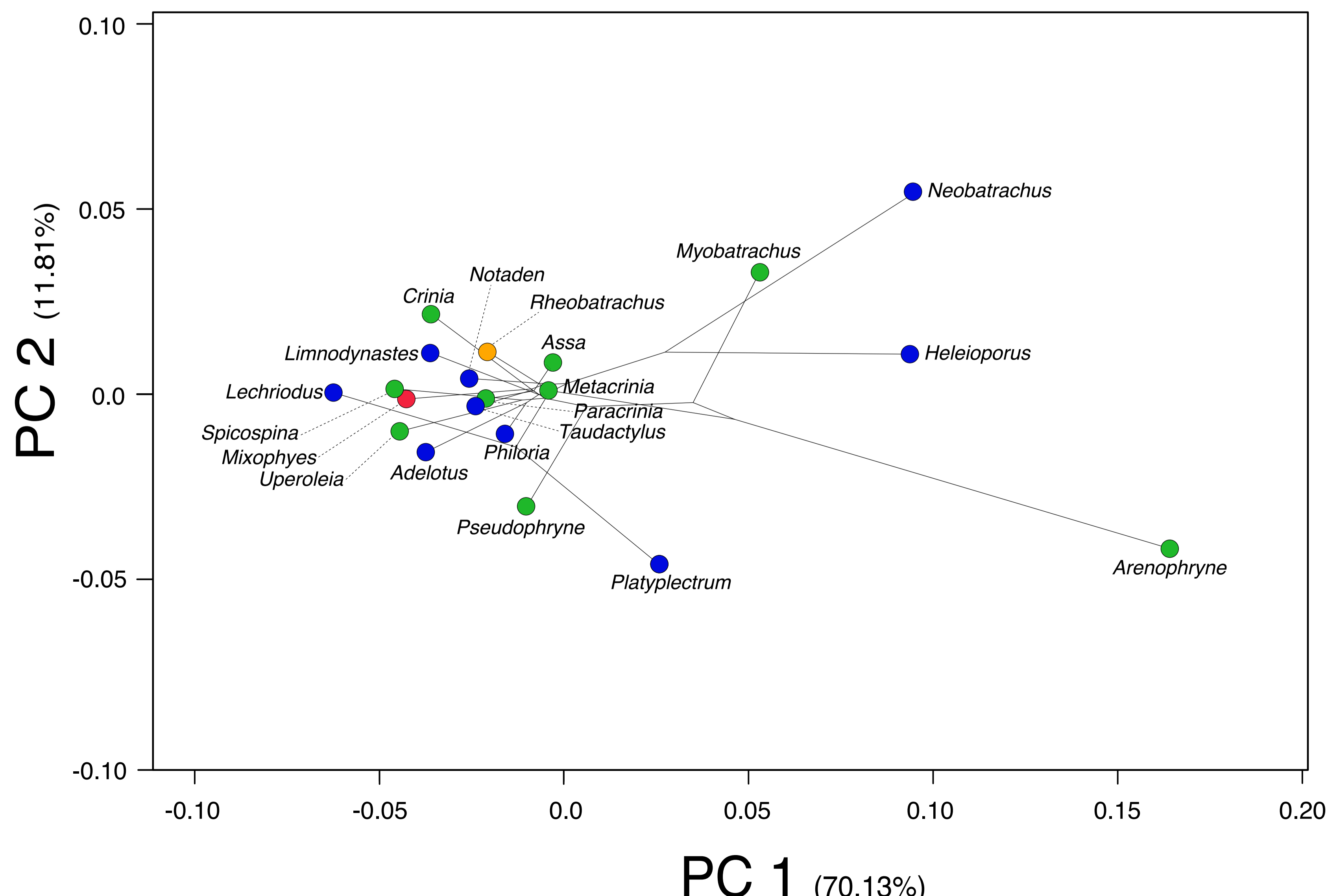

Supplement: Supplementary file 4 — (a) Phylomorphospace of PCA values on shape variation of radioulna (RU); (b) Phylomorphospace of PCA values on shape variation of tibiofibula (TF); (c) Phylomorphospace of PCA values on shape variation of humerus (H); (d) Phylomorphospace of PCA values on shape variation of femur (F); (e) Phylomorphospace of PCA values on fore-limb shape variation (RU + H); (f) Phylomorphospace of PCA values on hind-limb shape variation (TF + F). (PDF 332 kb) [file 12862_2017_993_MOESM4_ESM.pdf]

a

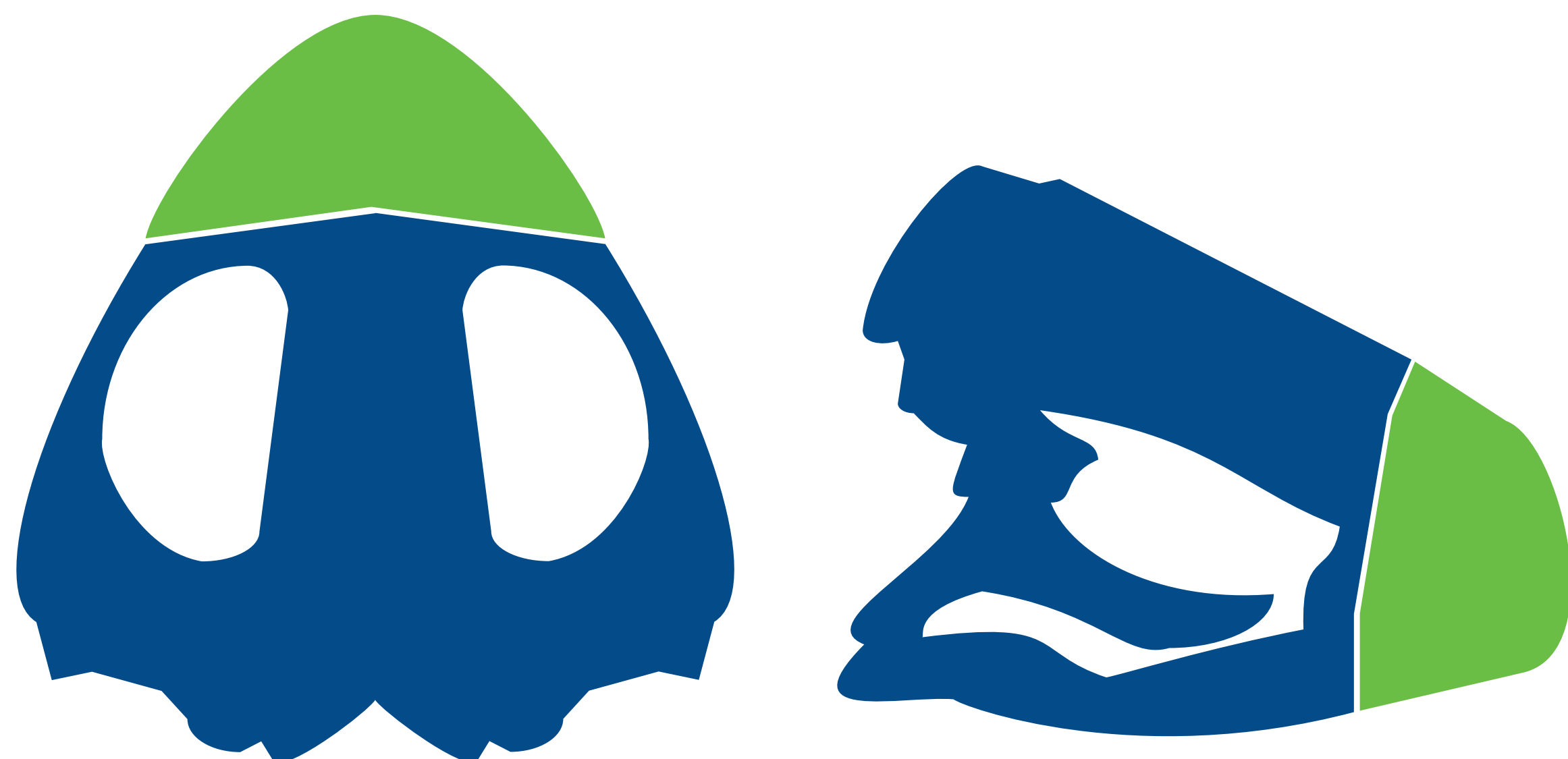

b

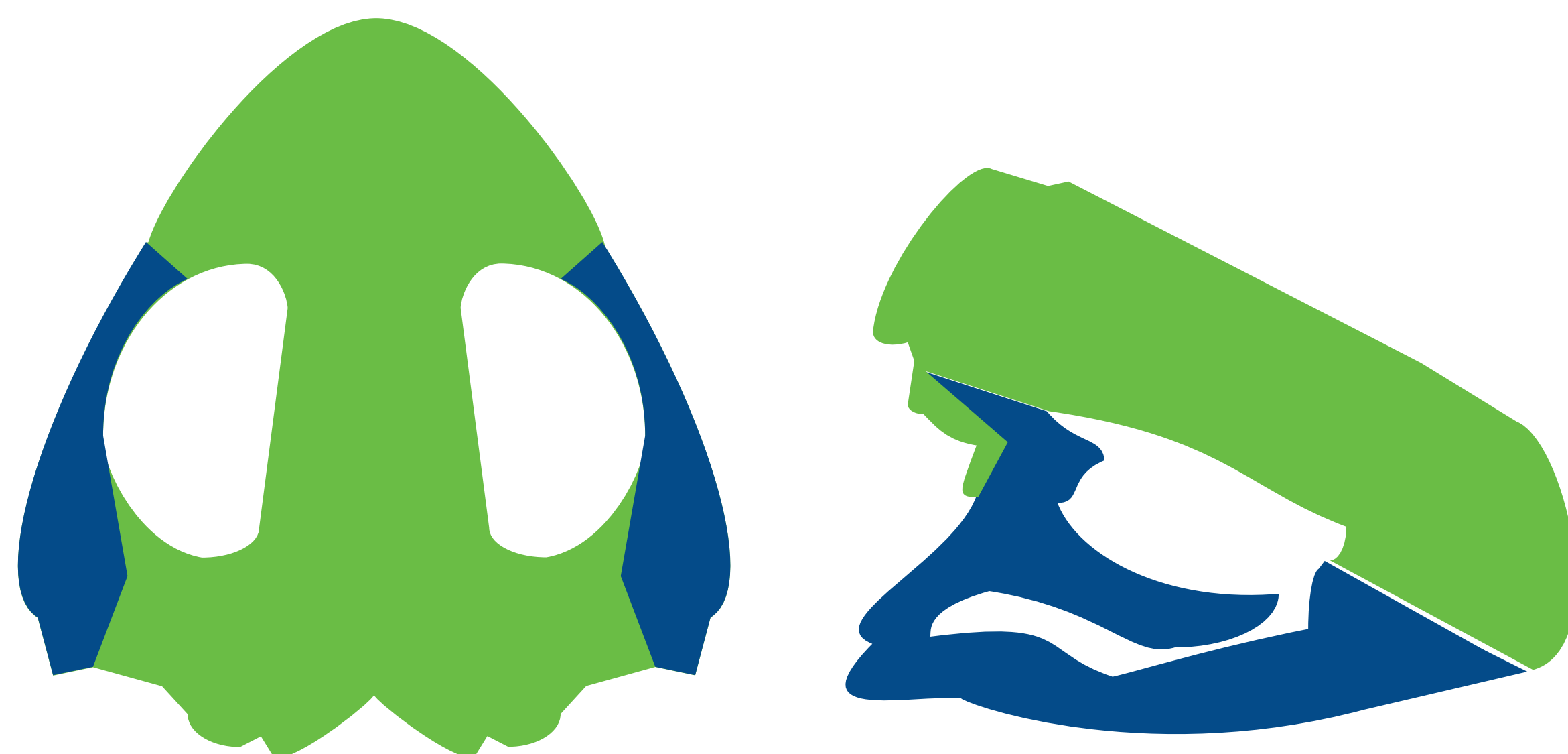

c

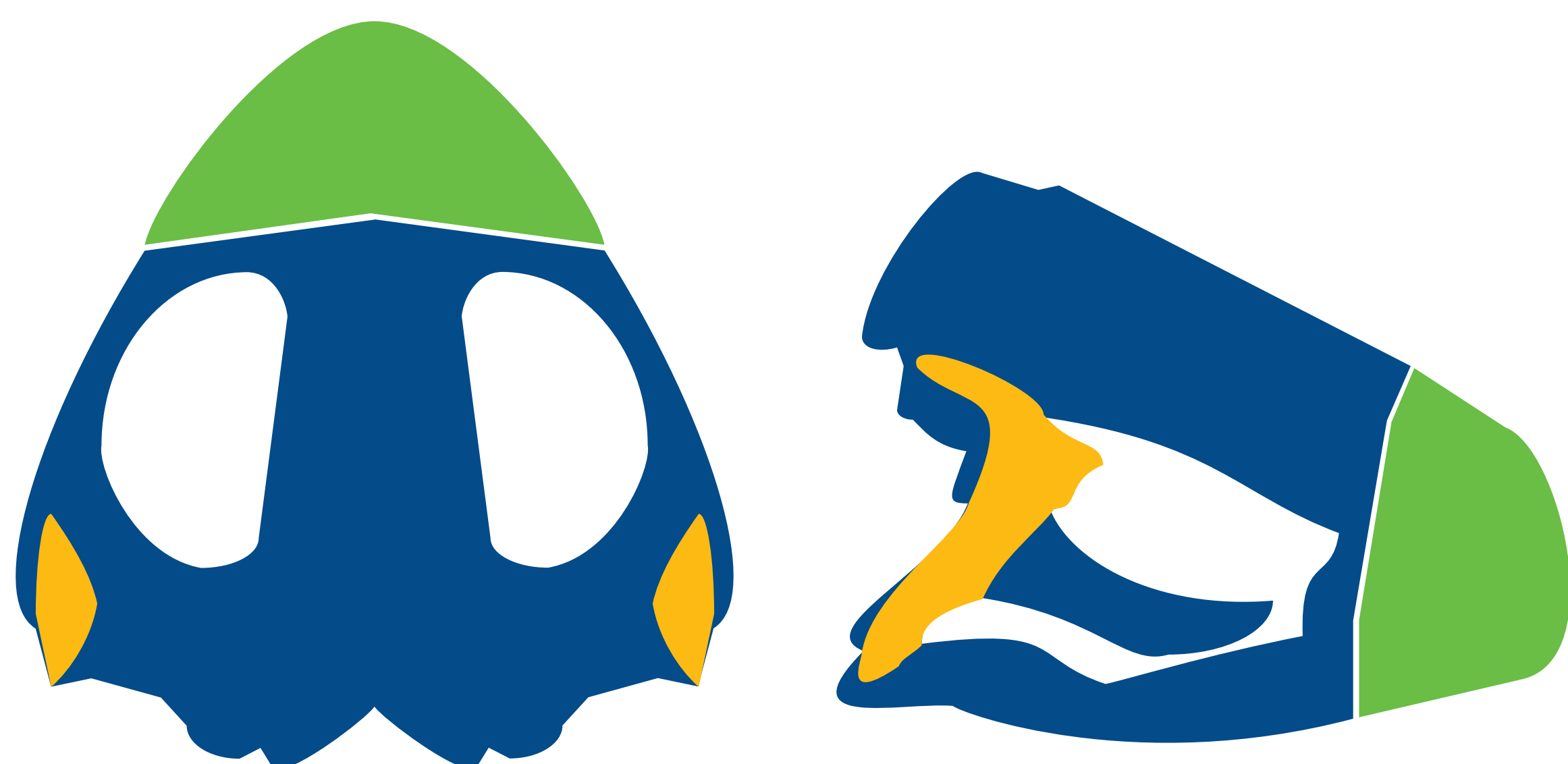

d

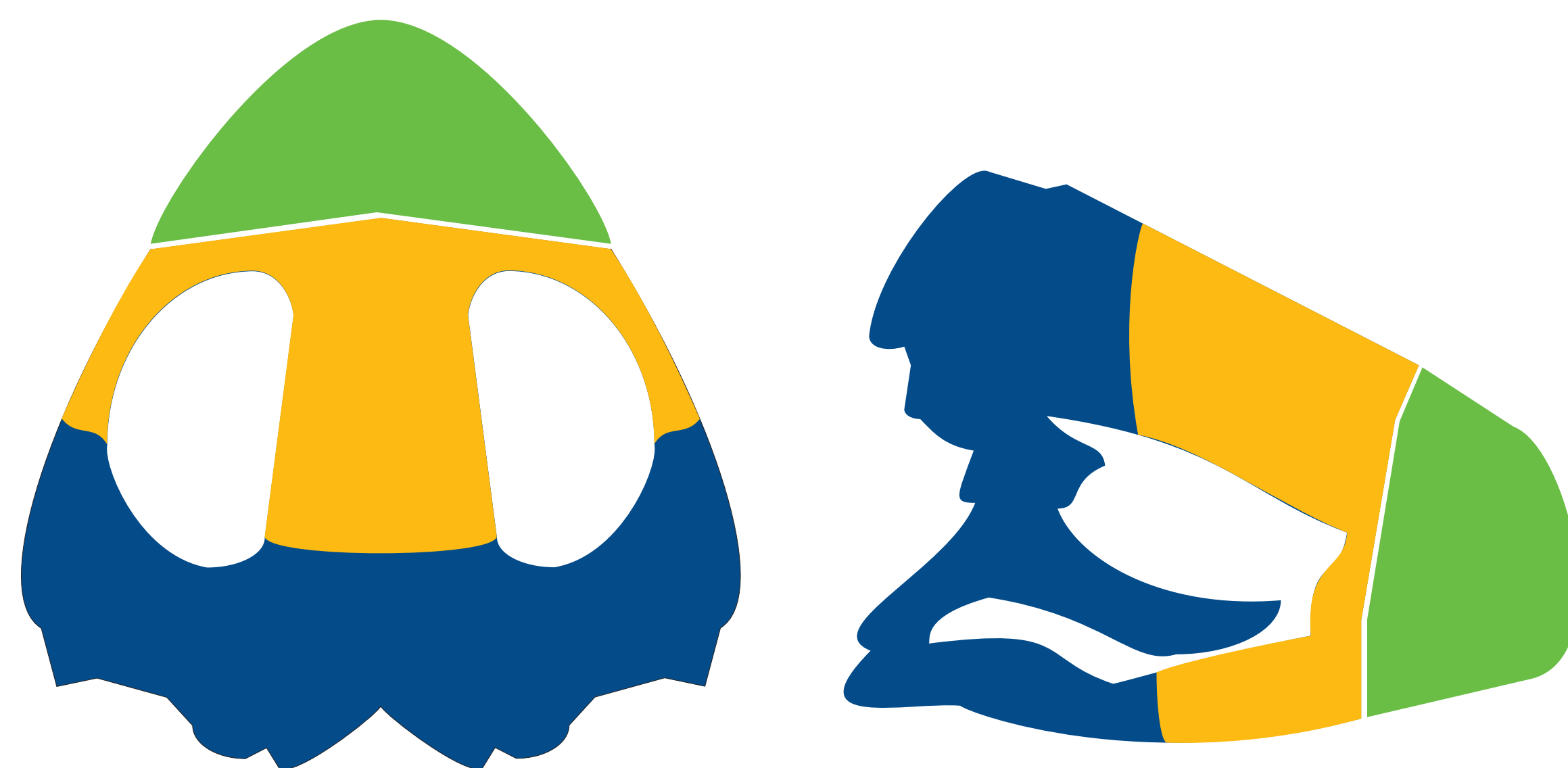

e

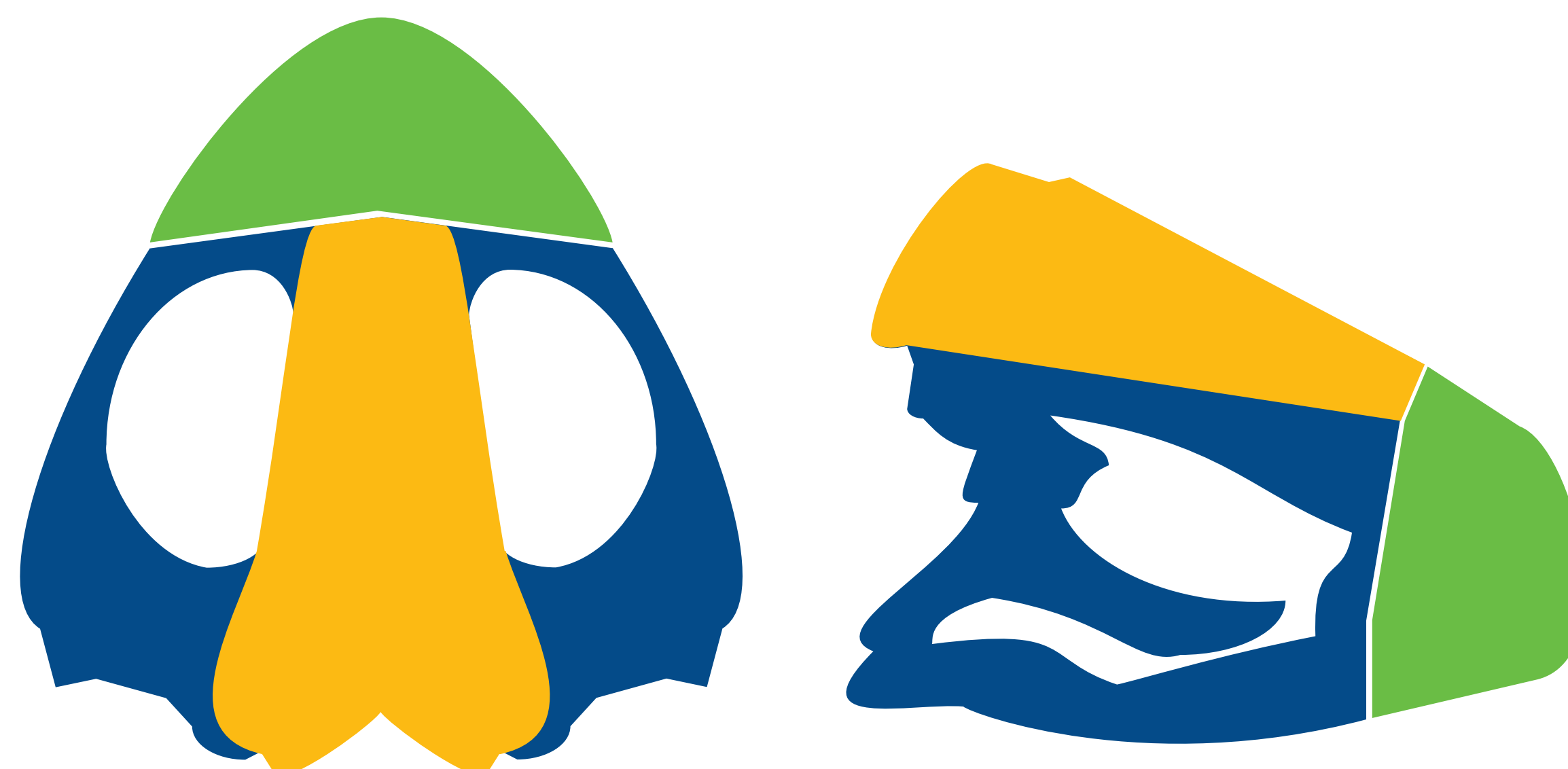

Supplement: Supplementary file 5 — Modular configurations modeled for the skull with two or three different partitions, based on different evolutionary hypothesis based on biological relevant regions. The different colours depict different modules. (a) The first module (green) includes the tip of the snout and the olfactory area (premaxilla, maxilla, and nasal), as it captures a lot of morphological variation among frog species, whereas the second module (blue) includes the rest of the skull; (b) this configuration captures skull depth – the first module includes the dorsal region of the skull, and the second module captures morphological information from the ventral region; (c) this tripartite model splits the skull in three modules: snout (green), squamosal (orange, which is part of the suspensory apparatus), and the rest of the skull (blue); (d) The first module depicts the snout (green), the second includes the medial part of the skull (orange), and the third module includes the most posterior region of the skull (blue); (e) this tripartite configuration includes a first module (green) with the snout morphology, a second module (orange) that encompasses the brain region (from the sphenethemoid to the exoccipital and foramen magnum, including the frontoparietal), and a third module (blue) for the rest of the skull. (PDF 275 kb) [file 12862_2017_993_MOESM5_ESM.pdf]
